# The Influence of the Hydroxyl Type on Crosslinking Process in Cyclodextrin Based Polyurethane Networks

**DOI:** 10.3390/gels8060348

**Published:** 2022-06-02

**Authors:** Cristian Peptu, Alexandra-Diana Diaconu, Maricel Danu, Catalina A. Peptu, Mariana Cristea, Valeria Harabagiu

**Affiliations:** 1 “Petru Poni” Institute of Macromolecular Chemistry, Aleea Grigore Ghica Voda 41A, 700487 Iasi, Romania; diaconu.diana@icmpp.ro (A.-D.D.); mcristea@icmpp.ro (M.C.); 2 Department of Natural and Synthetic Polymers, Faculty of Chemical Engineering and Environmental Protection, “Gheorghe Asachi” Technical University of Iasi, 71, Prof. Dr. Docent Dimitrie Mangeron Street, 700050 Iasi, Romania; mdanu@tuiasi.ro (M.D.); catipeptu@yahoo.co.uk (C.A.P.)

**Keywords:** cyclodextrin, cyclodextrin-oligolactide, polyurethane, polyethylene glycol, biodegradable hydrogel, isophorone diisocyanate

## Abstract

The influence of the hydroxyl groups (OH) type on the polyaddition processes of isocyanates represents a critical approach for the design of multicomponent polyurethane systems. Herein, to prove the effect of hydroxyl nature on both the isocyanate-OH polyaddition reactions and the structure/properties of the resulting networks, two structurally different cyclodextrins in terms of the primary and secondary groups’ ratio were analyzed, namely native β-cyclodextrin (CD) and its derivative esterified to the primary hydroxyl groups with oligolactide chains (CDLA). Thus, polyurethane hydrogels were prepared via the polyaddition of CD or CDLA to isophorone diisocyanate polyethylene glycol-based prepolymers (PEG-(NCO)_2_). The degradable character of the materials was induced by intercalating oligolactide short sequences into the polymer chains composing the polymer network. In order to establish the influence of the OH type, the synthesis of polyurethane hydrogels was analyzed by a rheological investigation of the overall system reactivity. Materials properties such as swelling behavior, thermal properties and hydrolytic degradation were influenced by the reaction feed. Specifically, the presence of primary OH groups leads to more compact networks with similar water uptake, disregarding the CD content, while the predominance of secondary OH groups together with the presence of oligolactide spacers leads to the fine tuning of the water swelling properties.

## 1. Introduction

Polymer networks composed of segmented polyurethanes were identified as good candidates for different applications, benefiting from excellent synthesis-properties correlations. Among the attractive traits of the crosslinked polyurethane networks, there may be mentioned the thermal and mechanical properties, biodegradability or biostability and biocompatibility. These properties are usually influenced by various synthesis parameters such as phase mixing, soft and hard segments chemistry, as well as the monomer/prepolymer content in the reaction feed, prepolymer molecular weight, etc. [1]. Typically, the hard segment mainly refers to the employed isocyanates, while soft segments are generally related to the polyether or polyester prepolymers. A special situation may be encountered for the carbohydrates-based polyurethane crosslinked systems used for, e.g., biomedical applications [2]. In the particular case of cyclodextrins, the polyols constitute hard segments and also contribute to the phase separation phenomena [3].

Cyclodextrins are cyclic oligosaccharides that have a torus-shaped ring structure, with a smaller rim, where primary hydroxyl groups are located, as well as a larger rim displaying secondary hydroxyl groups. The combination of cyclodextrins with other polymeric materials confers the ability to form host–guest inclusion complexes to the newly formed structures [4,5,6]. Applications that mainly use this property are related to the pharmaceutical field for entrapping and the controlled release of bioactive principles [7,8], alongside the environmental field in which it is beneficial for the purification of water polluted with various chemical agents [9], the food industry for the protection or extraction of active principles [10,11], or to chromatography [12] for separations based on host–guest interactions, among others.

The polyurethane-type structures based on cyclodextrins, of tight or relaxed networks, have been extensively studied for various applications, as described elsewhere [2,13,14]. Given the specific structure of cyclodextrins, with the anisotropy of the hydroxyl groups’ chemistry and their polar spatial distribution, one may expect certain influences over polyaddition reactions. Native cyclodextrins participate in polyaddition reactions with two types of hydroxyl groups, primary OH (OH–P) and secondary OH groups (OH–S). Understanding the influence of OH in multicomponent polyaddition reactions may be crucial for the design of complex polyurethane systems [15]. In principle, β-cyclodextrin (CD) has 21 OH functions divided into three groups according to the position of the neighboring carbon atom, at positions 2, 3 and 6 on the glycoside ring. The reactivity of each type of OH is different: the primary OH–C6 groups are the most reactive while the secondary OH–C3 groups are sterically hindered and the least reactive [12]. Moreover, the OH–P groups can freely rotate while the OH–S groups are relatively rigid. These differences in reactivity permit a certain selectivity in CD chemical modifications and dictate the behavior of various chemical reactions [16].

The current study takes into consideration the preparation of cyclodextrin-based polyurethane hydrogels with controlled biodegradable behavior and three types of building blocks expected to grant the obtained network specific properties. Thus, the polylactide (PLA) sequence provides hydrolytic degradability [17,18] and as a spacer influences the network architecture [19], while the polyethylene glycol (PEG) chains ensure the hydrophilicity and flexibility of the network [20], and the CDs act as network crosslinking moieties and imparts host–guest inclusion abilities [14,21,22]. The use of these three elements together may lead to the development of advanced materials comprising all the above mentioned properties. Combinations of PLA with PEG and CD have already been prepared through radical polymerization crosslinking [23] and herein, we investigate the possibility of preparing tri-component networks through the polyaddition reaction of alcohol groups to isocyanates.

Several applications were envisaged for the CD-PEG networks, such as tissue engineering, drug delivery, coatings, solid–solid phase changing materials, etc. [24,25,26,27,28,29] The above mentioned studies made use of commercially available, native or modified, cyclodextrins. Although several groups studied the synthesis of CD-PEG hydrogels by polyaddition reactions of cyclodextrins OH groups to PEG-isocyanate prepolymers, the separate contribution of OH–P and OH–S in the crosslinking reaction and their influence on the properties of the polymer networks remained uncharted [24,25,26,27,28,29]. Taking this into consideration, the current paper compares two crosslinking systems for the preparation of polyurethane hydrogels, using native and modified cyclodextrins with an altered OH–S/OH–P molar ratio in order to clarify the impact of specific cyclodextrin chemistry on the crosslinking behavior and the properties of the synthesized polymer networks.

Generally, researchers employ diisocyanates such as diphenylmethane diisocyanate (MDI) [27] or hexamethylene diisocyanate (HDMI) [24,25,26,28,29] as the urethane sequences in order to prepare PEG-diisocyanate prepolymers, which are also used in crosslinking with cyclodextrins. Herein, the isocyanate (NCO) component of the polyaddition system, isophorone diisocyante (IPDI), was used for various organic coatings [30] and also in the preparation of cyclodextrin materials due to its low crosslinking propensity [31,32,33,34,35,36,37]. One of the main reasons behind the frequent use of IPDI is related to the possibility of controlling the reaction process due to the particular chemical structure that endows the NCO groups with different reactivity at relatively low temperatures [38].

Another issue is related to the introduction of the oligoester sequences to cyclodextrin-polyurethane networks. Usually, oligoester prepolymers are employed and, e.g., CD-polyurethane-polycaprolacrone polymers were synthesized [39]. Our approach considers a prior step of cyclodextrin functionalization through the ring opening oligomerization of D,L-lactide [40,41,42] and its further use in polyaddition reactions as polyols.

## 2. Results and Discussion

**Scheme 1:**
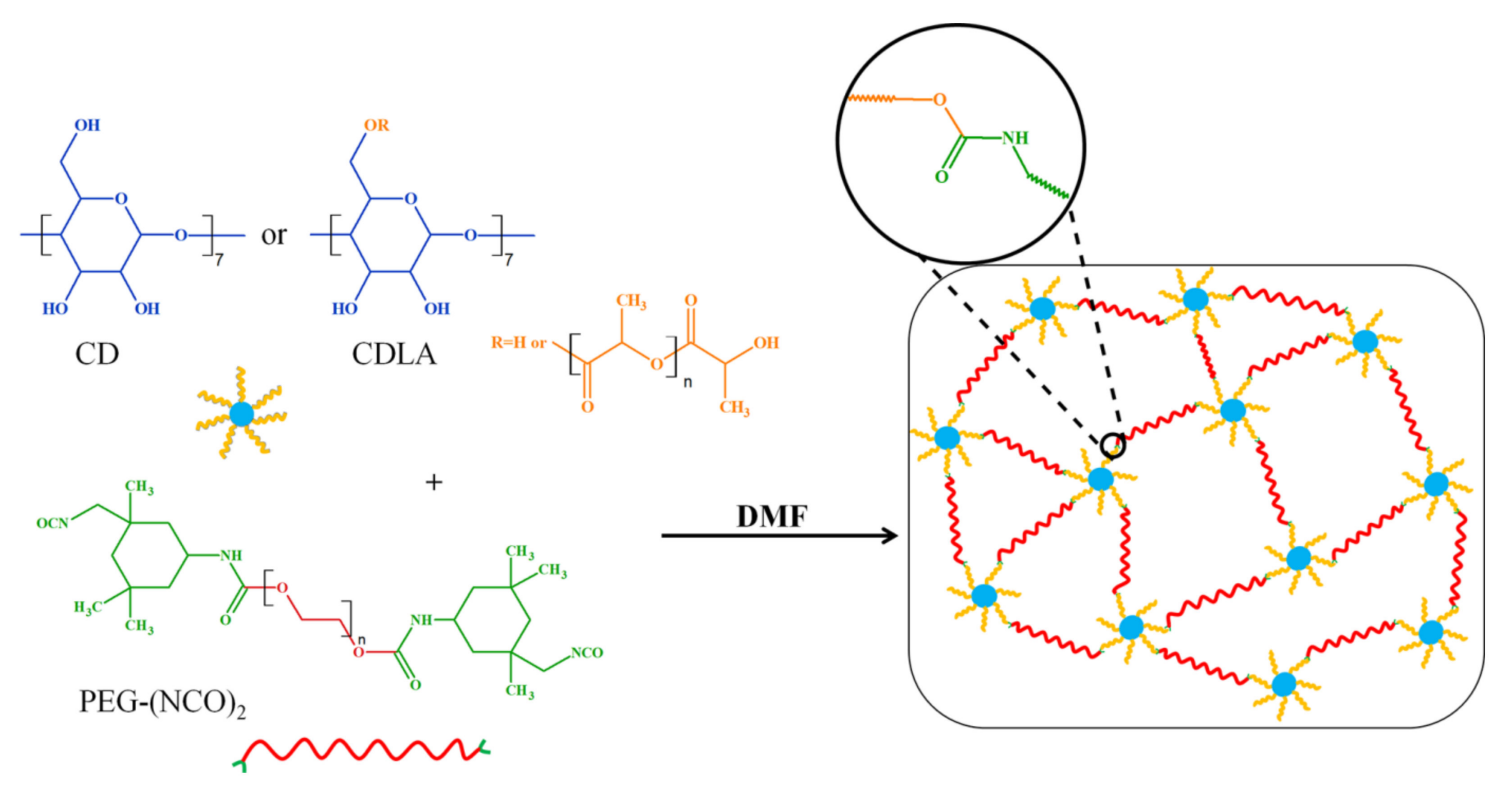
Synthesis of CD-PEG and CDLA-PEG hydrogels.

The OH–type influence on the outcome of the polyaddition reaction was studied by taking into consideration two different hydroxyl sources, namely native CD (containing 7 primary hydroxyl groups (OH–P) and 14 secondary ones (OH–S)) and a custom prepared cylodextrin-oligolactide (CDLA), with the OH–P groups being partially esterified. Thus, the evolution of the crosslinking reaction of either CD (B series) or CDLA (G series) with PEG-(NCO)_2_ (Scheme 1) was followed.

To be able to clearly comprehend the hydroxyl type effects, an accurate structural characterization of the starting prepolymers was performed, emphasizing the mass spectrometry structural characterization of the prepared products.

A bottleneck in the crosslinking of OH functional compounds with different reactivity (CD, PLA-diol and PEG-diol) in polyaddition reactions to isocyanates is that these three component systems may lead to heterogeneous bridges in the crosslinked networks. The reason for such inhomogeneity can be attributed to the reactivity differences between the hydroxyl groups: primary versus secondary groups, steric factors (e.g., placement of OH groups on the torus shape of CD) and different flexibility and mobility of polymer chains, e.g., PEG vs. CD. The tri-component reaction system also presents different miscibilities in the reaction solvent and variations in the diffusion coefficients and physical interactions among each other (e.g., the possibility to form pseudopolyrotaxanes with CD). Such physicochemical compatibility problems may be circumvented by introducing a supplementary reaction step, the covalent coupling of CD with PLA and PEG with IPDI, prior to the crosslinking reaction.

### 2.1. Prepolymers Synthesis and Structural Characterization

*Cyclodextrin-oligolactide (CDLA) prepolymer* was synthesized according to the literature [40,41] by covalent attachment of oligolactide chains to the β-cyclodextrin. The MALDI MS analysis assisted in calculating the *Mn* = 2443 g/mol and the dispersity index, *Ð* = 1.033. The *Mn* value was used for the calculation of the prepolymers molar ratios in further crosslinking reactions. As demonstrated in previous studies [40,41] by comparing MS and ^1^H NMR results, the oligolactide monomer units are connected to the CD as multiple chains (5 chains in average), with an average of three lactate units per chain, prevalently attached to the OH–P groups of CD, on the smaller rim. As a consequence, after native CD modification, the OH groups available for further crosslinking are mostly secondary (in average 19 OH–S out of 21), originating from oligolactide chain ends or CD unreacted groups. The altered OH–S/OH–P balance for CDLA vs. native CD has considerable importance for the crosslinking process, as further discussed.

*Polyethylene glycol-bis-isophorone diisocyanate (PEG-(NCO)_2_) prepolymers* are commonly employed for various polyurethane structures. PEG chains have OH–P groups at both ends, which can be conveniently modified with different reagents [43]. The reaction of polyols and IPDI proceeds slowly (e.g., the reaction between polypropylene glycol and IPDI in the absence of a catalyst lasts 111 h at a temperature of 80 °C) [44] and studies in the literature recommend the use of catalysts such as dibutyltin dilaurate (DBTL) [45] in order to benefit the NCO group’s reaction selectivity for a coupling process.

In order to control the PEG end chain functionalization with isocyanates, Cesteros et al. [25] monitored the polyaddition reaction in situ, using an FTIR probe. In this way, one may monitor the transformation of isocyanates without clear quantitative proof for the connectivity with the polymer. On the other hand, mass spectrometry has been used successfully to characterize the modified polypropylene glycol oligomers with IPDI [44] or the poly(ε-caprolactone)-diol modified with hexamethylene diisocyanate [46]. Consequently, in this work, the reaction between PEG and IPDI was evaluated using MALDI MS.

**Figure 1:**
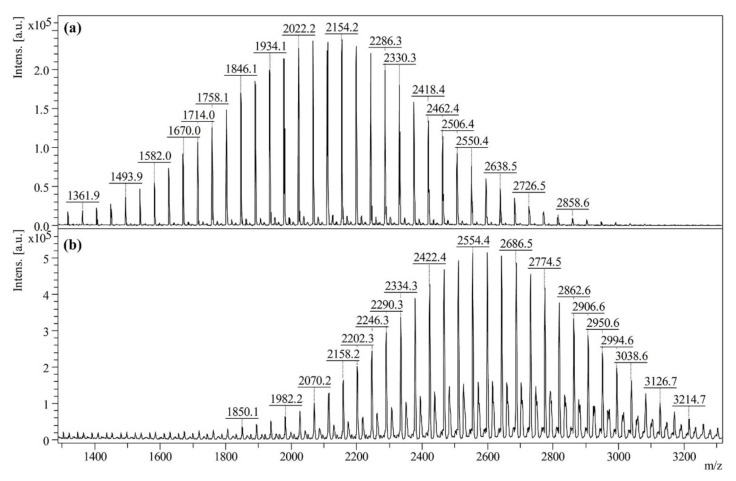
MALDI MS spectra of: (**a**) initial PEG, and (**b**) PEG-(NCO)_2_ product.

The functionalization of PEG with IPDI proceeds in DMF solution at 50 °C and leads to a PEG end capped with two isocyanate groups—PEG-(NCO)_2_ (Scheme S1, SI). In Figure 1a, one may observe the mass spectrum of the initial PEG, noting a Gaussian distribution of two series of signals in the mass range 1440–2900 centered at around *m*/*z* = 2154.2 [*2154.1 = 48 × 44.023 (ethylene oxide monomer units) + 18 (H_2_O) + 23 (Na)*—where the number of monomer units *n = 48*]. The higher intensity series corresponds to the distribution of the adduct species formed with Na cations [PEG + Na]^+^, while the less intense series corresponds to potassium adducts [PEG + K]^+^. For each series, the difference between two consecutive signals is 44 *m*/*z* units (corresponding to the monomer unit of ethylene glycol), and the difference between the signals of the two series is 16 *m*/*z* units (corresponding to the difference between the mass of potassium and mass of sodium). The MALDI MS spectrum helped to calculate the *Mn* of the PEG sample which was found to be *Mn* = 2125 g/mol and *Ð* = 1.023.

Comparing the MS spectra of the initial PEG sample and that of the sample taken from the reaction system after 30 min, in Figure 1a vs. Figure 1b, a right-shift of the MS peak’s Gaussian distribution is observed. For ease in analysis, we compared the *m*/*z* values of the base peaks from the MS spectra of the original PEG polymer and the PEG-(NCO)_2_ (*PEG* + 2 × *IPDI* equivalent with an Δ*m*/*z* = 2 × 222 between the corresponding chains having the same number of ethylene glycol monomer units) with a signal distribution in the mass range 1890–3240 (Figure 1b). Taking into account the peak with the highest intensity, in Figure 1b, having *m*/*z* = 2554.4 [*2554.4 = 47 × 44.023 (ethylene oxide monomer units) + 18 (H_2_O) + 2 × 222.13 (IPDI) + 23 (Na)*—the mass calculation formula of PEG-(NCO)_2_ where *n* = 47], it can be inferred that this spectrum confirms the synthesis of the desired product, PEG-(NCO)_2_ prepolymer. The obtained values of *Mn* = 2641 g/mol for the prepolymer indicate, however, a slight increase in the *Mn*, as compared with the theoretical value (*Mn*_theoretical_ = *Mn*_PEG_ + 444 = 2569 g/mol). This difference indicates a minimal sample fractionation, probably caused by secondary coupling reactions involving the PEG short chains. Additionally, a second peak series may be observed with lower intensity, at Δ*m*/*z* = 16, corresponding to the potassium-charged adducts [PEG-(NCO)_2_ + K]^+^.

The catalytic processes were found to favor chain elongation, thus affecting the results of the cross linking process [25]. However, the isocyanate groups of IPDI are not equivalent and have different reactivities towards OH groups [47], which can be used to diminish chain elongation reactions [48]. In fact, IPDI cycloaliphatic isocyanate groups were demonstrated to be 12 times more reactive than aliphatic groups towards primary OH groups in the presence of DBTL catalyst [47]; moreover, the isomeric *Z-E* forms of IPDI do not significantly influence the determined reactivity ratio. Therefore, in our case, after the addition of the PEG hydroxyl end groups to the secondary isocyanate group of the IPDI, the reduced reactivity of the remaining aliphatic NCO group resulted in a lengthier polyaddition process, ensuring that the multiple PEG chains connections were minimal.

### 2.2. CD- and CDLA-PEG Hydrogels

In order to compare the prepared hydrogels with similar materials and to isolate the crosslinking effect of the CDLA oligoesters, with an increased number of OH–S groups, we chose to also perform the reaction in the presence of neat CD. Hydrogels were obtained by the crosslinking reaction (Scheme 1) of native (B series) or modified CDs (G series) with the PEG-(NCO)_2_ product in the DMF solution at 50 °C, catalyzed by DBTL (various reaction parameters such as type of hydroxyl compound, CD or CDLA, and molar ratios between reactants may be found in Table 1).

After the reaction, these materials were subjected to a thorough purification treatment to remove the reaction solvent (DMF) and unreacted moieties by successive swelling in acetone and water followed by freeze-drying. The yields of the crosslinking reaction were over 90% for native CD and above 80% for CDLA, therefore suggesting that prepolymers were not fully reacted and the polyaddition process was slightly different for the considered systems. This was expected since our hypothesis states that the CDLA product participates in the crosslinking reaction mostly through OH–S functions, thus leading to slower polyaddition and possibly faulty network formation, as observed by others for different gelation systems employing OH–S functions [15]. Moreover, DBTL catalyzes the isocyanate reaction with OH–P much more effectively than it catalyzes the reaction of isocyanate with OH–S [49].

**Figure 2:**
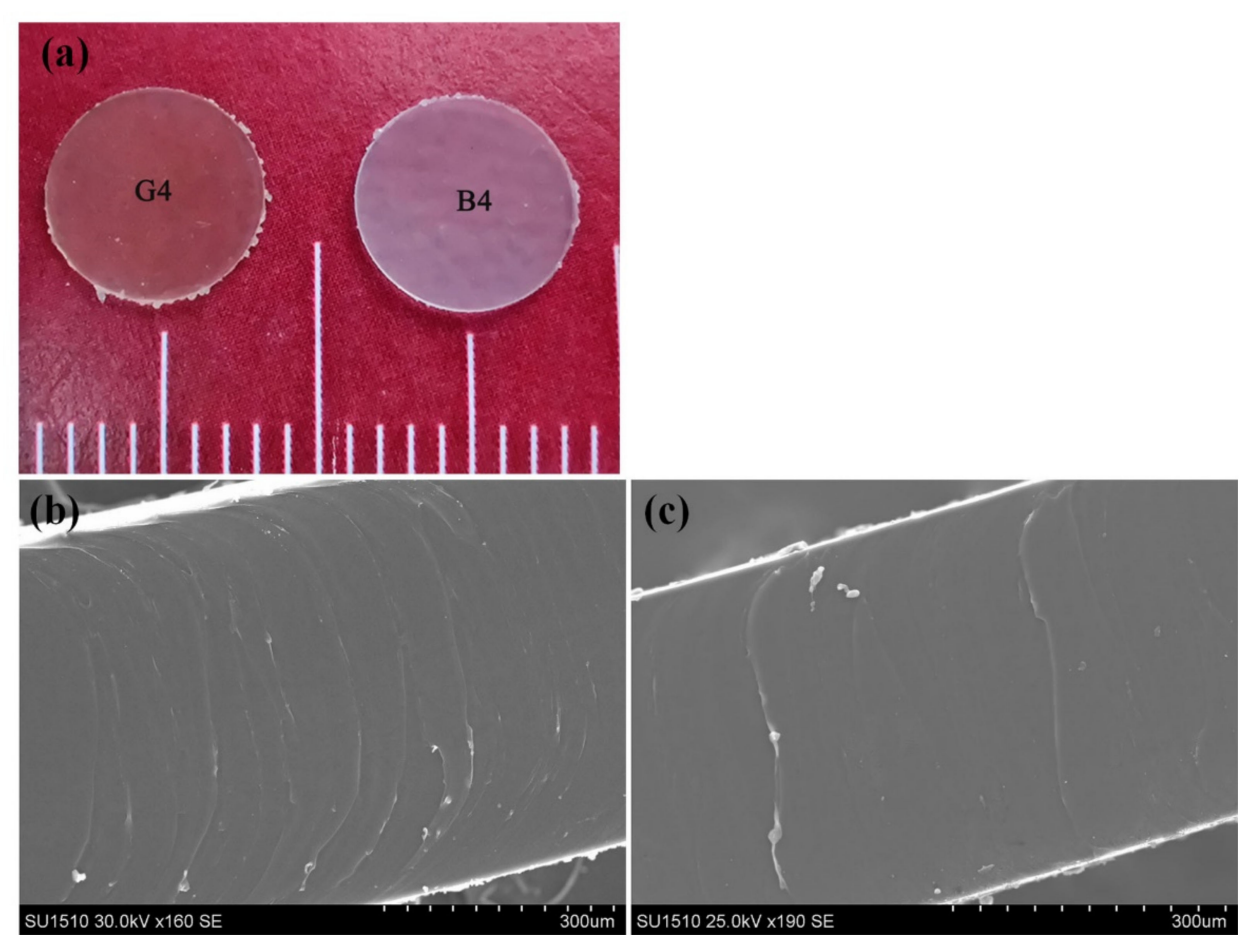
Description of CD-PEG and CDLA-PEG hydrogels (**a**) visual and SEM micrographs (**b**) B4 and (**c**) G4 samples.

The morphology of the obtained hydrogels (after purification) depends on the particular conditions of the crosslinking reaction, and, in the case of CDLA crosslinked polymer networks, the obtained materials are homogeneous and somewhat more transparent than the hydrogels obtained using native CD, which are slightly opaque, as can be observed from the photograph presented in Figure 2a. The macroscopic differences are probably related to the presence of the oligolactide component and, also, to the physical and chemical interconnectivity differences between native CD and CDLA within the crosslinked network. This may also be correlated with the solubility changes induced through the functionalization of cyclodextrin with oligolactides [41,42] which may impact the course of the crosslinking.

Scanning electronic microscopy micrographs (Figure 2b,c), performed on the hydrogels cross-section, reveal that samples are homogeneous, without macro-pores or any noticeable morphological differences between the B and G samples.

The integrity of the hydrogels resulting from the crosslinking reactions was found to depend on the molar ratio between CD or CDLA and diisocyanate PEG prepolymers. Previous studies demonstrated that the increasing amounts of cyclodextrin lead to inhomogeneous crosslinking between CD and PEG-diisocyanate [24,25,26,27,28,29]. To obtain the mechanically stable hydrogels, the limit of the molar ratio between CD and PEG-(NCO)_2_ was found to be close to 1:1 for both hexamethylene diisocyanate (HDMI) and methylene diphenyl diisocyanate (MDI) [26,27,29]. In our work, the hydrogels, with molar ratios between CDLA and PEG-(NCO)_2_ of 1:1 and 1:2 (entries #1 and #2—Table 1) in the reaction feed, had an inhomogeneous aspect, associated with a faulty crosslinking process. Thus, for G1 and G2 preparations, a certain viscosity of the reaction mixture was observed, but the mechanical stresses during samples handling led to mechanical ruptures of the hydrogel. This can be explained on the basis of the excess OH groups, which lead to network structural defects, as the insufficiency of the isocyanate groups hinder the formation of interconnected three-dimensional networks in the whole reaction mass. In fact, most of the previous studies concerning the preparation of cyclodextrin-PEG polyurethane networks employed an excess of PEG-diisocyanate in order to ensure a homogeneous crosslinking [24,25,26,27,28,29]. Similarly, in the case of polytetramethylene glycol(PTMG)/IPDI/CD networks, the highest value for the CD/PTMG molar ratio was also established at 1/4 [33].

The limitations of successful crosslinking may also be related to the type of diisocyanate employed, because of its specific reactivity. The IPDI is less reactive than, e.g., HMDI, thus affecting the crosslinking reaction. Moreover, the isocyanate groups of IPDI have different reactivities [48] and, in fact, the PEG-(NCO)_2_ prepolymers have a lower reactivity than the initial IPDI towards OH groups because the more reactive NCO groups (cycloaliphatic) are consumed during prepolymers formation. Additionally, the CDLA contains more OH–S groups than CD and, consequently, has a lower reactivity towards NCO groups.

**Figure 3:**
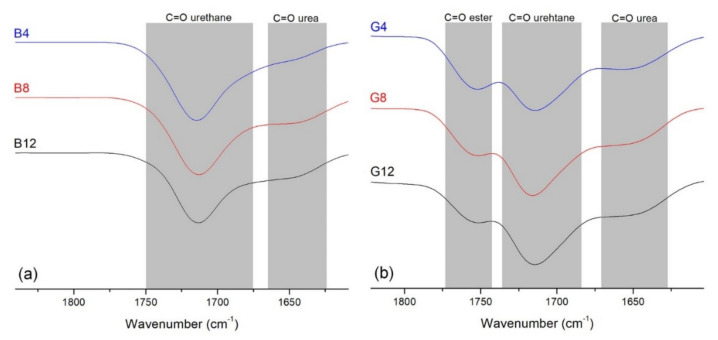
Highlights of FTIR spectra: (**a**) CD-PEG, and (**b**) CDLA-PEG.

The purified hydrogels were evaluated by using FTIR in order to observe the disappearance of isocyanate groups and the formation of the urethane connections to cyclodextrin derivatives. The band specific to the –NCO functional group (2270 cm^−1^) is absent from all spectra (Figure S2) while the formation of polyurethane bonds is supported by the observation of polyurethane specific bands: –NH at 3568 cm^−1^, –CO at 1715 cm^−1^ and –NH–CO at 1539 cm^−1^). Additionally, it may be observed that unreacted isocyanates were quenched during the water purification step as suggested by the band observed around 1650 cm^−1^, specific for urea carbonyl (C=O) bonds (highlighted in Figure 3). The appearance of the urea characteristic bands may be correlated with the water quenching of the unreacted NCO groups, formation of amine groups and their conversion in urea upon their reaction with another isocyanate function. Such processes may occur during slow curing processes or purification procedures. Thus, in the case of the B series, the C=O urea band fades as the amount of CD increases. On the other hand, in the FTIR spectra of the G series, the band corresponding to urea is more distinct, signifying that a greater number of isocyanate groups remained unreacted, possibly due to a less effective crosslinking process. Additionally, residual water from cyclodextrins, which is not fully removed prior to the polyaddition reaction, can contribute to urea formation [50].

As a specific feature of the G series, the presence of the band associated with the ester C=O bond (1730 cm^−1^) in oligolactide, is visible in Figure 3. The overlapped spectra (see also Figure S2), demonstrate that the amount of carbonyl groups from ester functions (the band at 1750 cm^−1^) decreased from the G4 to G12 samples; this signifies that the oligolactide amount in each sample is proportional to the amount of CDLA added in the reaction feed.

### 2.3. Dynamic Rheology Investigation of the Crosslinking Reactions

Taking into consideration the observed differences between the crosslinking reactions in the presence of CD and CDLA, we decided to investigate the gelation process through an in situ dynamic rheological analysis. Thus, the influence of the OH type and of the molar ratios between hydroxyl compounds and diisocyanates on the outcome of the crosslinking reaction was investigated using as a general parameter the gelation time, and rheological measurements. The viscoelastic quantities measured in situ, during the evolution of the dynamic rheological properties of the hydrogels which were formed in the coupling reaction between the isocyanates and OH groups, allowed for a precise evaluation of the employed reaction systems.

**Figure 4:**
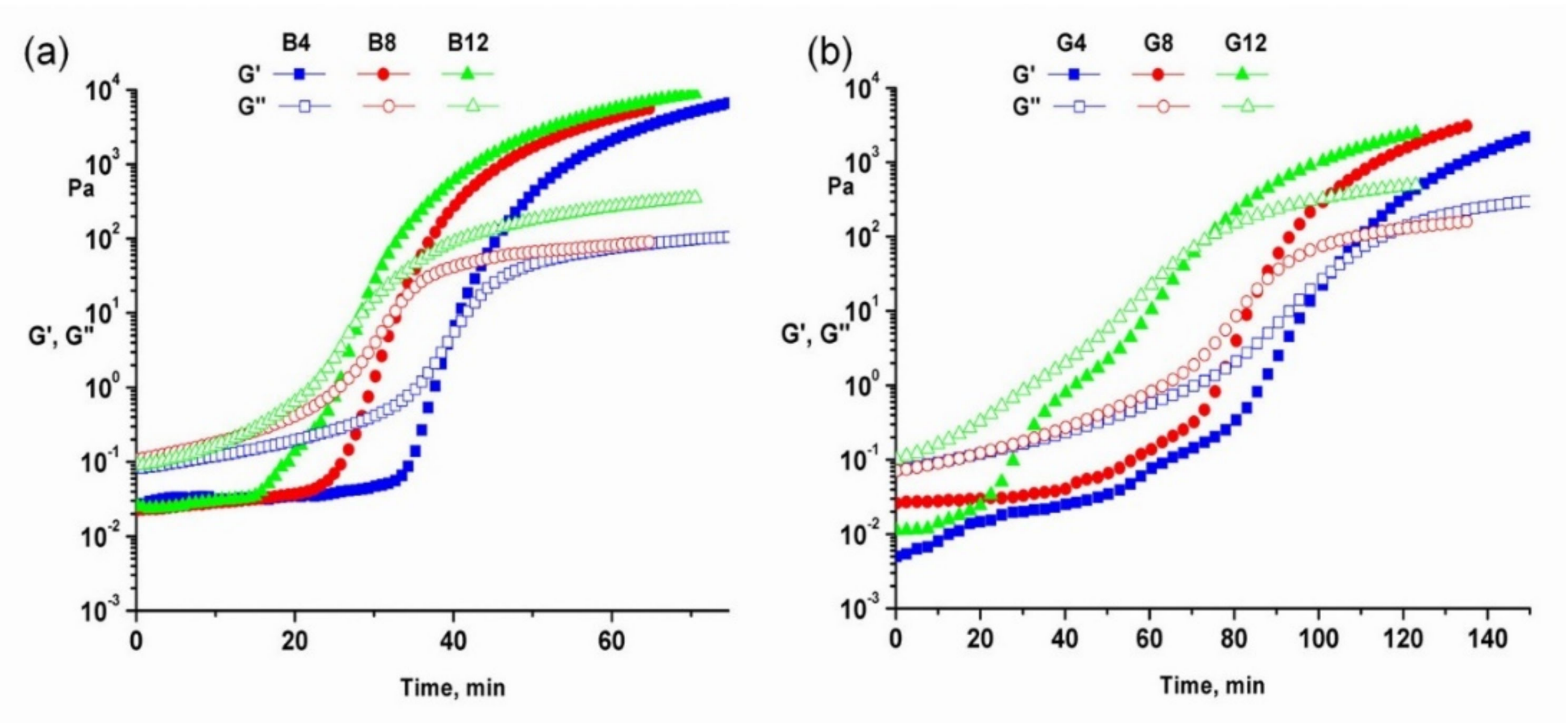
Comparative time sweep rheological test of (**a**) CD-PEG and (**b**) CDLA-PEG hydrogels syntheses.

The rheological profiles of two series of crosslinking systems obtained for hydrogels with neat cyclodextrins and CDLA are presented in Figure 4. The kinetic measurements allowed us to determine the gelation time, the moment at which gelation of the reaction system begins—defined as the crossover point of the viscoelastic moduli, storage modulus (G’) and loss modulus (G”), respectively. The evolution of the G’ and G’’ parameters begins in a typical liquid state (G” > G’) for the hydrogels crosslinked with CD. Further, after specific periods of time, the reaction mixture undergoes gelation processes signaled by the steep increase in G’, accompanied by a moderate increase in G”. The crossover point observed during the G’ and G” evolution is consistent with the gelation point (G’ = G”) and represents the onset of the crosslinking reaction [51] which is correlated with a transition process from a viscous to a solid-like behavior, specific to the tested frequency. A clear variation of the characteristic gelation time with the content of reacting species in the initial feed may be observed (Table 2 and Figure 4). Thus, the gelation time (T_x_, where x nominates the hydrogel sample) increases with the amount of CD, as follows: T_B12_ < T_B8_ < T_B4_. This behavior may be explained by the fact that, for mixtures with a lower content of OH groups, a shorter period of time is needed for oligosaccharide incorporation within the three-dimensional network and also for the gelation of the respective formulation via polyaddition reactions. Similar behavior was observed for the reaction systems containing CDLA (G series), as gelation proceeds faster when there is a lower content of CDLA in the reaction feed (T_G12_ < T_G8_ < T_G4_). Therefore, no matter the type of CD crosslinker, an excess of isocyanates leads to faster OH consumption and, consequently, to a faster polyaddition process.

However, when comparing the B and G series, for similar molar ratios between the total number of hydroxyl and isocyanate groups, the G series gelation times are significantly higher. For example, in the case of G12, the gelation begins much later than B12 and this fact is consistent for all the considered pairs in the G/B series. The main difference between the compared reaction systems is related to the nature of the OH groups. Thus, OH–S groups are more predominant in the G series, thereby resulting in decreased overall reactivity. Although during the crosslinking process of B samples one may expect the involvement of OH–S groups from the larger rim of CD, the onset of the crosslinking reaction is more likely to occur at the expense of the OH–P groups. However, in the case of CDLA derivatives, their structural characterization by NMR [41] revealed that functionalization with oligolactide oligomers occurs at the smaller rim, thus changing the nature of the available OH–P (native CD) into OH–S groups (oligolactide end chains). Moreover, the oligolactide grafts on the CD may sterically hinder the remaining primary OH–P groups. Therefore, the mentioned structural differences between the cyclodextrins used for the B and G series lead to a different crosslinking behavior. The structural differences between the OH sources are also correlated with the presence of a DBTL catalyst which is less effective in catalyzing the NCO reaction with OH–S [49]. Moreover, according to the work of Tilly et al. [15], when studying the influence of the OH type on crosslinking via isocyanate polyadditions, one may expect the crossover time to increase with the content of OH–S groups in the reaction feed. Their study showed that the apparent gelation energy needed for the crosslinking of systems containing more OH–S groups than OH–P is higher, thus explaining the differences observed herein between the B and G series. Additionally, in their study, parallel FTIR and rheology kinetics demonstrated that the different OH–S content in the reaction feed leads to changes in the monomer conversion at gelation point which, ultimately, leads to the different properties of the polyurethane networks. Similarly, we may expect different properties of the G and B series derived from the alteration of the OH–P/OH–S balance in the initial reaction feed.

### 2.4. Thermal Properties

**Figure 5:**
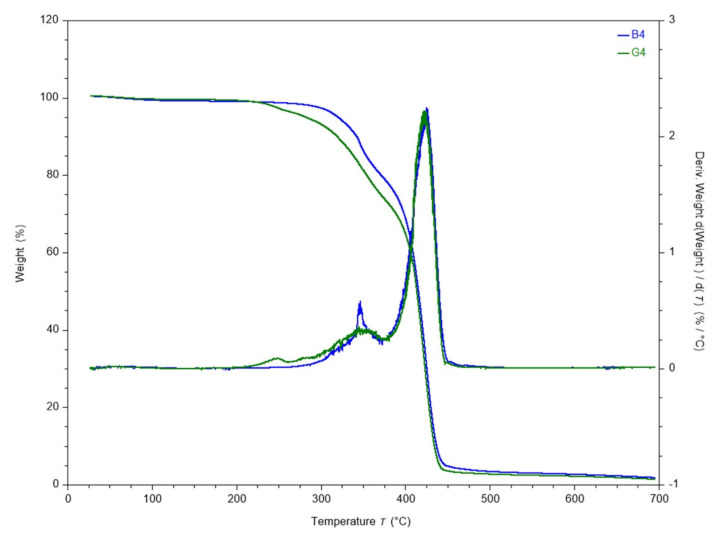
Overlapped thermal degradation curves for the B4 and G4 hydrogel samples.

The thermal degradability of the prepared polyurethane networks is correlated with the component sequences (description in SI section, Figures S3 and S4—thermal degradation of CDLA and PEG). Both hydrogel series, B and G, are characterized by two main thermal degradation steps (Table S1, Figures S5 and S6). However, G series onsets with a less significant step, correlated with the presence of the oligolactide sequence, which shifts the overall onset of degradation to lower temperatures (see T5 values in Table S1). Moreover, the weight loss after each degradation step is higher for G samples. This behavior is exemplified in Figure 5, which compares the degradation behavior of the B4 and G4 samples. Given the structural difference between the B and G samples, this behavior can be attributed to the particular features of the G type networks, such as the incorporation of the lactide or less connectivity between CDLA and PEG chains.

The major step of degradation associated with the zone 220–370 °C reflects overlapping degradation events, such as decomposition of oligolactide chains, cyclic oligosaccharides and cleavage of urethane linkages (Figures S5 and S6). The sharp drop in weight that begins at around 375 °C corresponds to the degradation of PEG segments and of advanced fragments produced during previous decomposition steps. The behavior in this region does not depend visibly on the presence of oligolactide sequences. When the pairs B4-G4, B8-G8, B12-G12 are compared, it appears that there is no systematic change in thermal stability versus composition in such complex systems as the thermal stability is often governed by the environment of the given groups [52].

**Figure 6:**
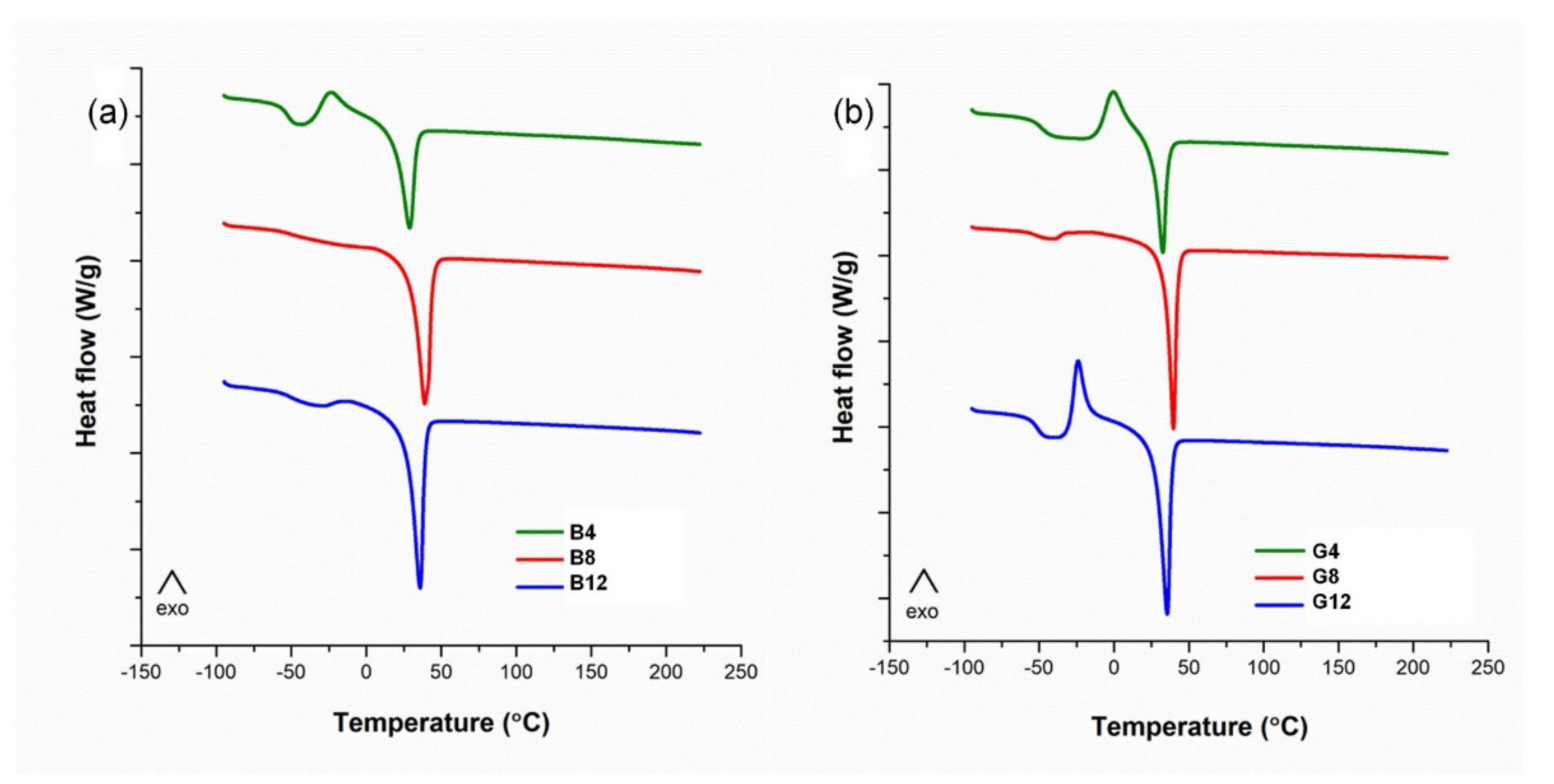
Differential scanning calorimetry analysis of (**a**) CD-PEG and (**b**) CDLA-PEG hydrogels.

The recording of the DSC characterization data presented in Figure 6 was performed during the second heating stage. The main thermal characteristics obtained from DSC are outlined in Table 3.

Generally, after the glass transition temperature the samples present an exothermic peak, associated with the crystallization of the amorphous domains, and an endothermic peak that corresponds to the melting of the crystalline domains. This is characteristic for both the B and G series. As the PEG/CD molar ratio increases from 4 to 8, the mobility of the network increases. The samples B8 and G8 crystallize during cooling and they do not present any crystallization in the heating step. This aspect is also sustained by the smaller values of ΔCp of B8 and G8 as compared to the B4 and G4, which indicates the presence of crystalline domains at the beginning of the second heating. The amorphous regions are constrained to a higher degree by the crystalline domains for the samples B8 and G8. However, as more PEG is included in the networks (B12 and G12), the mobility of the networks and the ability of crystallization during cooling is again reduced. This effect is more evident for the G series.

An interesting feature of CD-PEG networks is related to their capacity to undergo solid–solid phase transitions which may be used for energy storage materials [27]. The variation in the cyclodextrins content in the polymer networks, either CD or CDLA, affects the melting enthalpies (Table 3). Previously, studies were conducted on polymer networks of PEG with a molecular weight of 8 × 10^3^ g/mol [26]. Herein, due to the lower *Mn* of PEG, the CD/PEG molar ratio led to a greater influence over the weight proportion between PEG and CD for both the B and G series. Thus, the melting enthalpy values increased significantly with the increase in PEG content. Although the B and G series have a similar CD/PEG molar ratio, the theoretical PEG weight ratio is higher for the B series. Assuming that the prepolymers ratio in the reaction feed is largely maintained in the obtained polymer networks, we may observe that the melting enthalpies of the G series are lower than in the case of their B series homologues. However, higher enthalpy values are associated with higher degrees of freedom for the soft PEG chains, which is equivalent to lesser connectivity in the polymer network [27]. Therefore, we may consider that in the case of the B series crosslinking, the presence of OH–P groups prevents the further involvement of the remaining OH–S groups due to steric hindrance and finally leads to a lesser connectivity of the PEG chains. On the other hand, for the G series, most of the OH groups are of the OH–S type, with lower reactivity, thus allowing a more homogeneous crosslinking and ensuring a better connectivity of the PEG in the network, with lower degrees of freedom, despite the slower crosslinking process.

### 2.5. Water Uptake Properties

**Figure 7:**
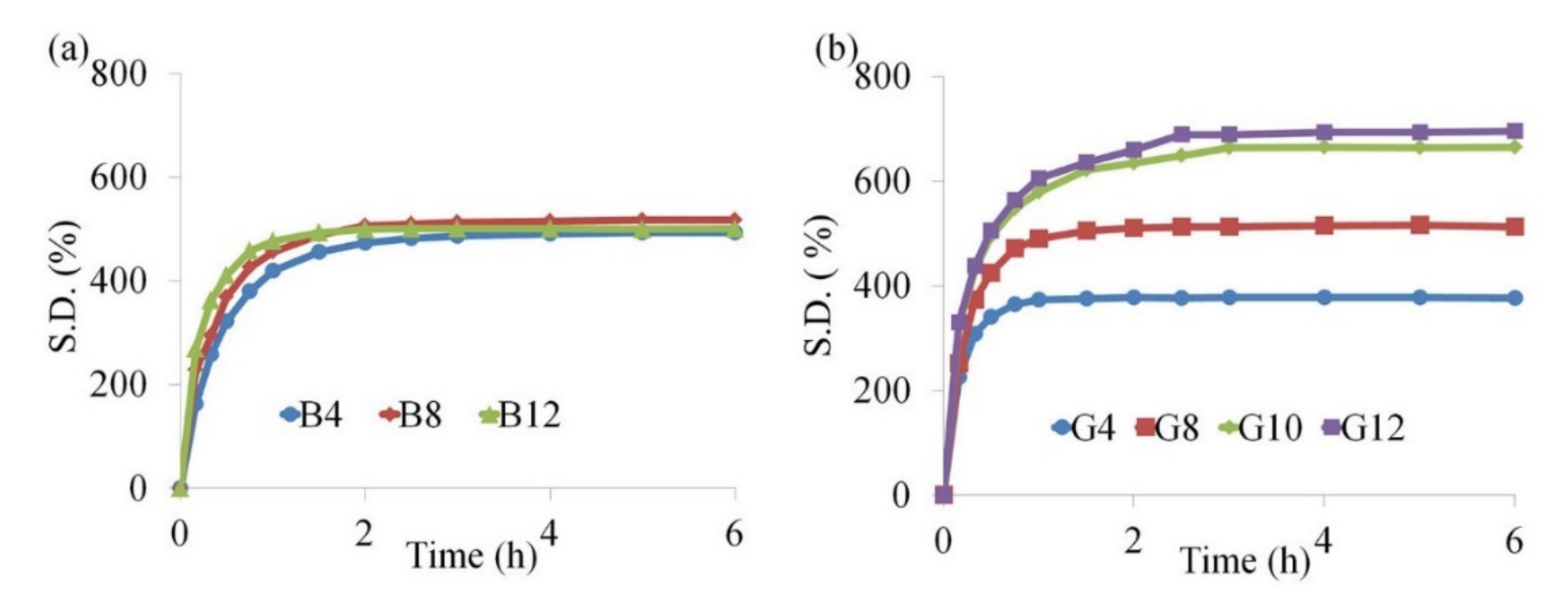
Swelling degree of (**a**) CD-PEG and (**b**) CDLA-PEG hydrogels.

The water uptake of the hydrogels from the B and G series is represented in Figure 7. Both types of hydrogels are characterized by a fast-swelling kinetics, reaching the maximum degree of swelling depending on the polymer network structure/composition.

The amount of CD does not greatly influence the degree of swelling for the B series (Figure 7a), as there are relatively small differences in the water uptake, no matter the CD content. The observed results are consistent with those previously published in the work of Cesteros et al. [24].

The low swelling effect of the CD content in the reaction feed, apparent in the B series, may be correlated with the crosslinking behavior observed in the rheological time sweep. Thus, the high reactivity of the OH–P groups leads to the rapid crosslinking and formation of tightly packed network regions with high amounts of cyclodextrins. This crosslinking behavior leads to the formation of PEG networks of relatively similar densities and swelling properties, disregarding the CD content in the reaction feed, in the studied CD/PEG molar ratios range. The decreasing content of CD leads only to a relatively faster swelling kinetics due to the increased weight ratio of PEG. The studies of Peng et al. [27] are in good agreement with our observations. They investigated the effect of increasing the CD amount in the reaction feed for β-cyclodextrin/4,4-diphenylmethane diisocyanate/polyethylene glycol crosslinked networks, with approximately similar CD/PEG molar ratios as in the current work. They evidenced, through polymer network crystallinity studies, that an increased CD feed leads to an increased degree of freedom for PEG chains. In fact, they inferred that only the most reactive OH groups of the CD partake in the crosslinking reaction namely the OH-P functions and that increasing the CD amount leads to similar tightly packed networks and a higher number of PEG chains with an increased degree of freedom.

On the other hand, the G series is characterized by a different swelling behavior (Figure 7b), as the decrease in CDLA content in the reaction feed leads to a clear increase in water uptake, from 350% (G4) to 700% (G12). Additionally, for the samples with a higher amount of PEG, G10 and G12, the swelling equilibrium requires more time to establish.

The presence of oligolactide sequences may influence the water uptake directly, being hydrophobic [53], and indirectly, by increasing the flexibility of the polymer network. The water uptake of G4 is lower than that of B4 due to the additional content of oligolactide chains that increase the overall hydrophobicity of polymer networks. Thus, the increased weight percentage of CDLA leads to a significant decrease in water uptake. However, when the samples G8 and B8 are compared, the water uptake is similar, showing that the effect of additional oligolactide sequences is precluded by the polymer network architecture. A further decrease in the CDLA feed determines a significantly higher water uptake for G12 sample as compared with its homolog, B12. Therefore, we inferred that the overall water swelling behavior of CDLA-PEG hydrogels is subject to the antagonistic influence of the oligoactide sequence, on one side by reducing the water uptake due to its hydrophobic character and, on the other by increasing the water uptake through the effect on the network architecture and flexibility.

The observed water uptake tendency of G networks may be correlated with the increased homogeneity of the nature of OH groups, in the crosslinking feed, leading to a more regular packing as compared with B networks. In fact, the crosslinking of the G series is a slower process that leads, finally, to more homogeneous networks with the swelling properties directly influenced by the CDLA amount in the reaction feed. Thus, the uniformity of the OH type (OH–S for CDLA) leads to more relaxed networks as compared with the B series, with a strong influence over the water swelling behavior. In the case of the B series, the predominance of OH–P groups during crosslinking determines network architectural changes that circumvent the CD weight ratio effect over the water uptake, as explained earlier.

Comparative studies, performed only in the case of the B4 and G4 samples (Figure S8, SI), revealed that water uptake decreases by more than 200% for both samples upon heating from 4 to 40 °C. The dependence of the swelling behavior of CD-PEG hydrogels on the temperature was previously observed in the studies of Cesteros et al. [24,25] and will be studied in future work.

### 2.6. Hydrolytic Degradation Study

**Figure 8:**
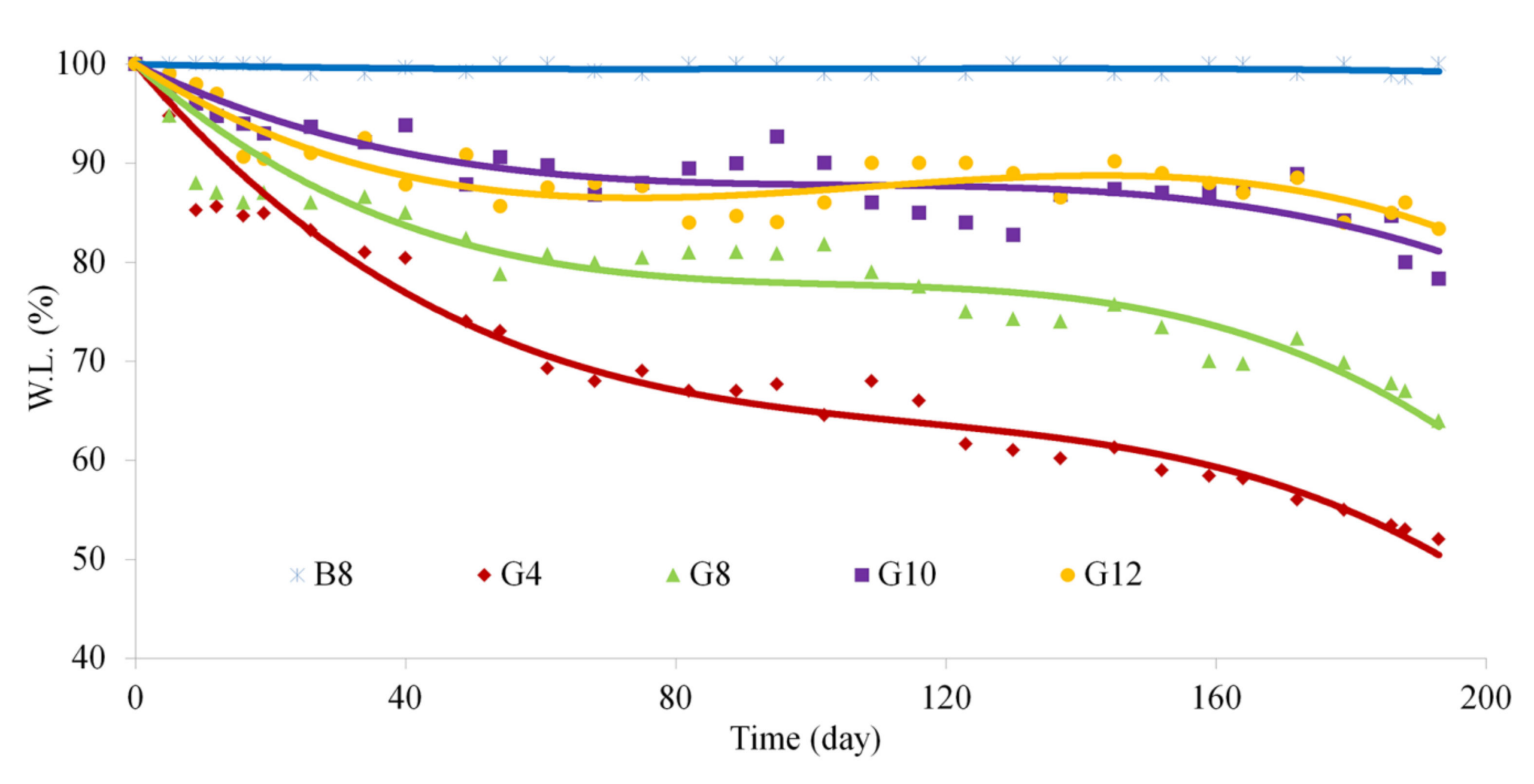
Graphical representation of mass loss due to hydrolytic degradation.

The introduction of the oligolactide sequence in the crosslinked networks provides them with their hydrolytic degradation capacity. The weight loss following the hydrolytic degradation of the CDLA-PEG hydrogels is presented in Figure 8 together with the weight loss measured for one CD-PEG hydrogel, used as non-degradable reference in the present degradation conditions (PBS buffer at pH = 7.4 and 37.5 °C). The reference sample did not suffer any significant weight loss while the G series experienced weight losses according to the oligolactide content in each sample. Therefore, the degradation pathway of the prepared hydrogels is mediated solely through the hydrolysis of the ester bonds, as expected. The highest weight loss may be noted in the case of the G4 hydrogel, of about 48% after 193 days. Samples with a lower oligolactide content (G10, G12) reached only an approximately 20% weight loss.

Additionally, it may be remarked that degradation has two phases for all G hydrogels—a rapid degradation in the beginning followed by a less steep region. The first phase lasts approximately 9 days for G4 and G8, and almost double this time for G10 and G12. This behavior indicated the possibility to control the degradation speed using adapted CDLA content in the synthesis feed. It is likely that rapid degradation occurs at the expense of the more accessible OLA-PEG chains which are partially connected to the polymer network, on one side. Additionally, oligolactides or even CDLA molecules with a low network connectivity may be also lost during this early phase. The second phase, which is less steep, involves the slow decay of network connections with a diffusional elimination of the water soluble components.

At this point, the discussion may be focused on the connectivity of the G type polyurethane networks and, more precisely, on the participation of the oligolactide hydroxyl end chains in the polyaddition reactions. Thus, a polymer network formed without polyaddition processes involving oligolactide end chains would lose only the polyester part via hydrolysis, which theoretically ranges from approximately 10 wt% (G4) to 4 wt% (G12) (Table 4). However, the G4 sample, which has ~10 wt% of oligoester, loses more than 15% of its weight after only 10 days. Additionally, the remaining G series homologues lose more weight than the oligolactide content, as may be observed by confronting the data from Table 4. Therefore, we may infer that the loss in PEG chains contributes to the observed degradation due to a significant connectivity with the oligolactides. This finding confirms that oligolactide end chains (OH–S) are involved in the network connectivity and correlates well with the previous observations of the time sweep rheology tests that showed a low reactivity of CDLA crosslinking due to the involvement of OH–S groups.

Additionally, hydrogels with a low CDLA content show a reduced weight loss in spite of the fact that their water uptake is higher which, in principle, favors faster degradation. If the network connectivity predominantly constituted OLA end chains, the hydrogels would rapidly disintegrate, which is not the case. Therefore, for a low CDLA content or in the presence of excess isocyanate groups in the reaction feed, we may assume that oligosaccharide OH groups (predominantly OH–S from positions 2 and 3) participate to a significant degree in the formation of urethane bridges which, in turn, are responsible for maintaining the integrity of the hydrogels under degradation.

In principle, the degradation of PLA-PEG networks does not only depend on the cleavage of the ester bonds but, also, on the physical structure of the polymer network [54], and more specifically on the possible phase separations that may occur during the crosslinking process. Although the initial SEM inspection of the hydrogels did not reveal structural defects related to different network packing or phase separations, further investigation of the degradation process may illuminate structural differences. The degradation process may be correlated with the homogeneity of the chemical connectivity, in the studied polymer networks, by inspecting the microstructure of the degraded samples.

The degradation process was followed using an SEM analysis of the degraded samples (freeze dried after extraction from the incubation chamber) at various stages of degradation, after 9, 89 and 193 days, respectively. The micrographs presented in Figure S8 revealed that the surface of the B8 reference sample is not significantly modified after 193 days (as expected from the weight loss experiment) while the G4 sample surface presents high porosity, specific to the polylactides bulk degradation [55].

**Figure 9:**
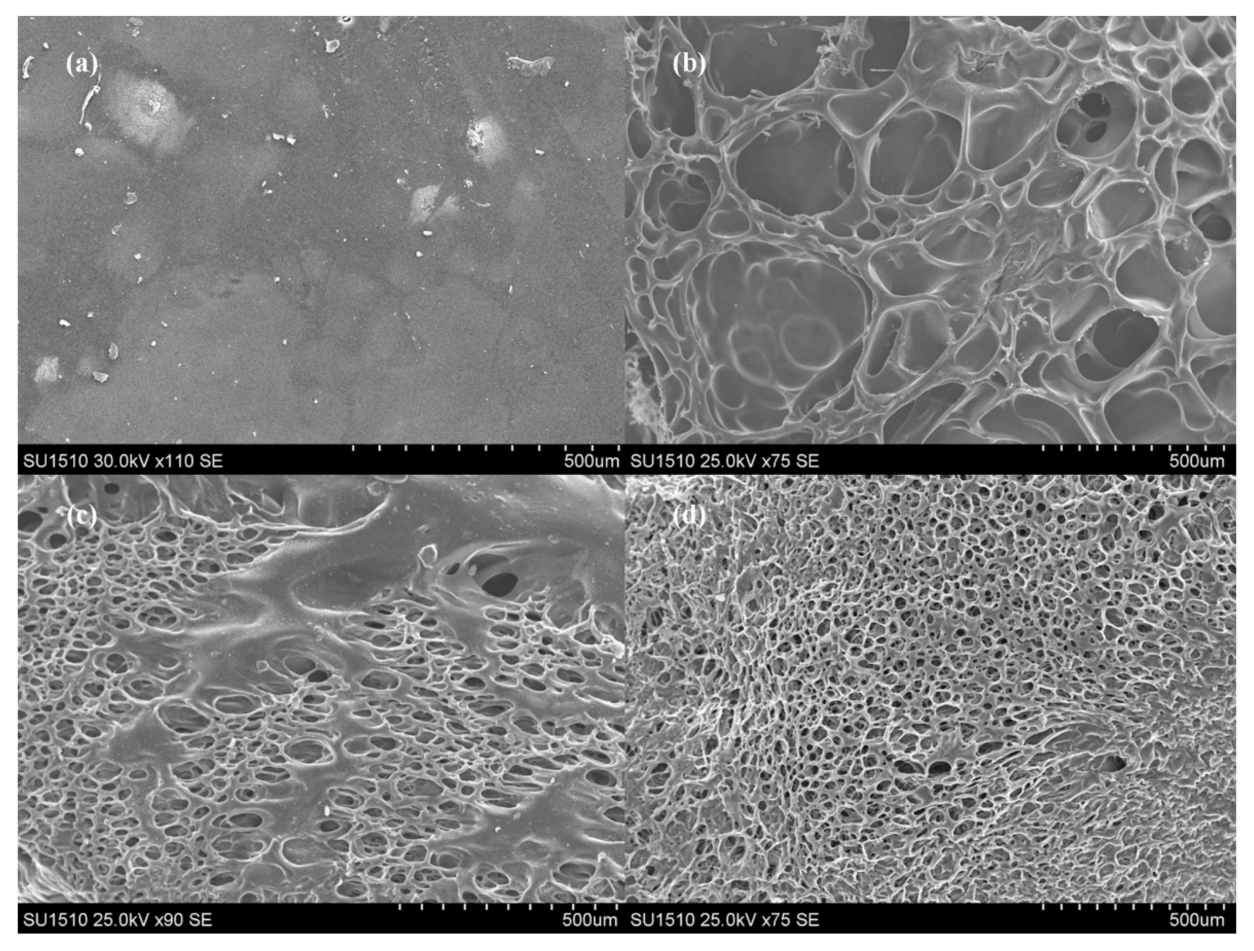
Surface micrographs of G4 sample: (**a**) initial, (**b**) after 9 days, (**c**) after 89 days, and (**d**) after 193 days of degradation.

The formation of pores was correlated with the weight loss profiles by analyzing the SEM images of the samples after specific degradation periods (Figure 9). Thus, after 9 days, large pores formed, with high dimensional distribution, ranging from 500 to 50 µm. At 89 days, the dimensional distribution of the pores narrowed greatly and some less porous hydrogel regions were observed. This demonstrates that, in fact, the G4 hydrogels have structural heterogeneity due to irregular crosslinking.

The SEM micrograph of the samples collected after 193 days of degradation reveal a relatively small reduction in the pores size, but the non-porous areas were completely eliminated as the hydrogel became entirely porous. The pores diameters of the final G4 degradation samples ranged from 50 to 5 µm (Figure 9d and Figure S9a,b). Although the porosity was relatively homogeneous, indicating bulk degradation, the pores regions with different orientations indicate macro-structural defects.

The specific porosity was influenced by the CDLA content as can be observed in the comparative micrographs of the G4-G8-G10-G12 after 193 days of degradation (Figure S9). Thus, the pore size is increasing as the amount of oligoester is reduced, indicating a proportionally diminished loss of material. Thus, the previous weight loss results concerning the influence of the CDLA content over the degradation rate are in good correlation with the SEM observations.

**Figure 10:**
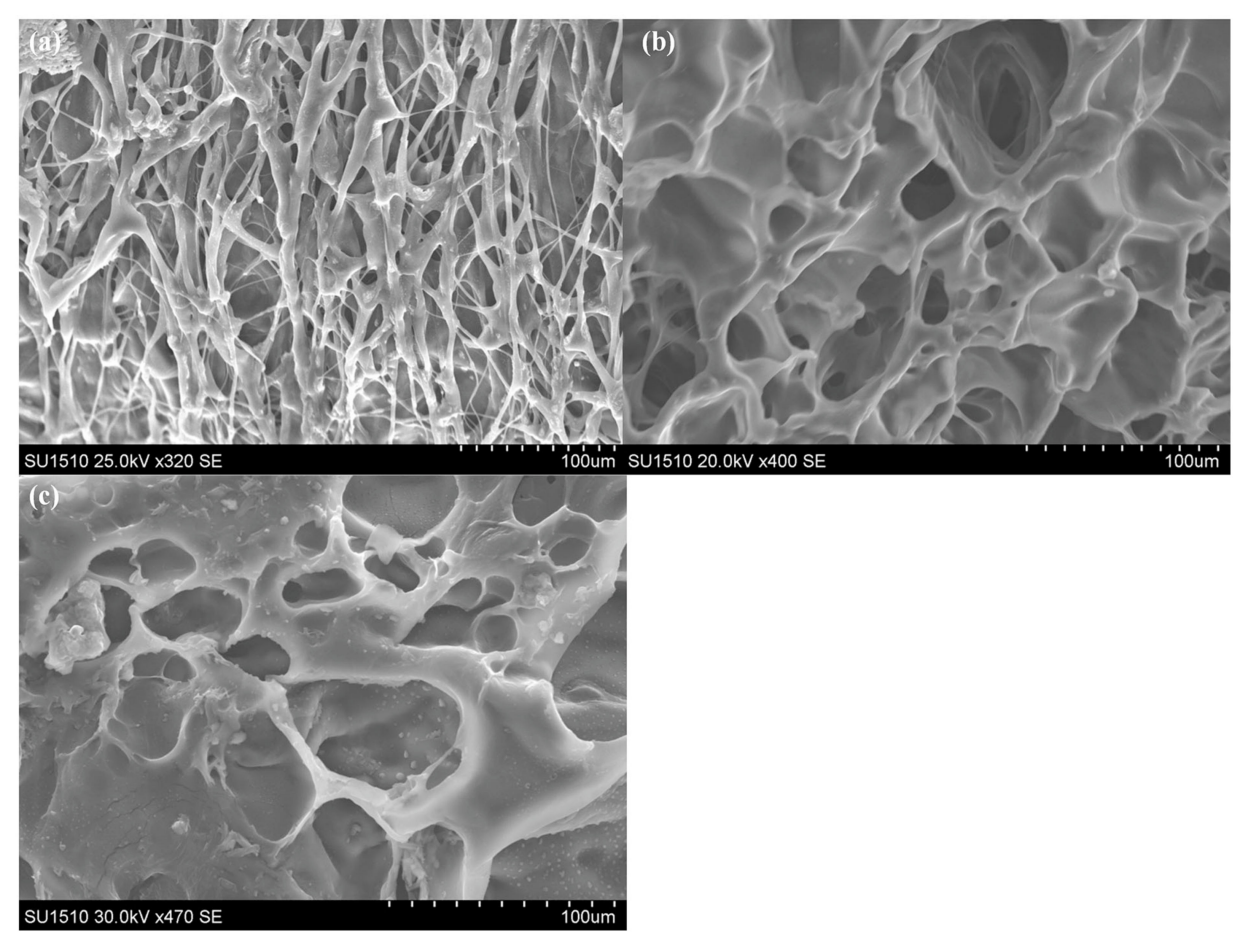
Micrographs of G series, section view, after 193 days of degradation: (**a**) G4, (**b**) G8, and (**c**) G12.

Besides surface examination, the fractured surface of the degraded hydrogels was investigated in order to obtain information about the progress of degradation in the sample volume (Figure 10). In the case of G4, cross-sectional intercommunicating pores was observed, indicating bulk material degradation in the entire sample volume. The porosity diminished for G8- and G12-degraded hydrogels, as the content of CDLA was lower, similar to the surface analysis. The observed degradation behavior indicates the potential of the prepared hydrogels for use as biomaterials for tissue engineering or drug delivery devices.

## 3. Conclusions and Perspectives

Our work emphasized the importance of the OH type in isocyanate polyaddition processes and especially in the case of cyclodextrin OH sources. Thus, the presence of more OH groups in the crosslinking feed leads to an increase in the gelation time as demonstrated by the in situ rheological measurements. A similar effect was observed when primary OH groups were replaced with secondary ones. Overall, we noticed that cyclodextrin-induced crosslinking is significantly affected by the nature of OH groups not only through direct observations, which were based on the investigation of the crosslinking process, but also indirectly, by analysing the effects on the materials properties. The water uptake of the prepared hydrogels revealed significant differences between CD-PEG and CDLA-PEG materials related to the amount and the nature of the OH groups. More specifically, the use of native CDs comes with the disadvantage that the swelling properties of the obtained hydrogels are not correlated with the CD feed in the employed reaction conditions. On the other hand, the predominance of OH–S, as in the case of CDLA, leads to a certain correlation between swelling properties and the reaction feed. Additionally, the water uptake of both types of hydrogels was diminished by the increase in temperature. Therefore, the prepared hydrogels will be further tested for potential applications as thermosensitive drug delivery materials. The thermal characterization of the prepared CDLA-PEG networks revealed solid–solid melting transitions similar to those of CD-PEG. The synthesis properties correlations were also observed at the level of the hydrolytic degradability of the CDLA-PEG type hydrogels. Thus, while CD-PEG type networks do not degrade in the employed conditions, the presence of oligolactides determines hydrolytic decay, as implied by the gravimetric measurements. Moreover, the porosity evolution during degradation processes observed using SEM images revealed a similar behavior as for polylactide-based materials, proving the incorporation of oligolactides in the polymer network via polyaddition at the level of end-chain OH–S groups. These results suggests CDLA hydrogels to be contenders for potential applications as skin regeneration scaffolds due to their hydrophilicity, drug loading capacity through physical inclusion in the cyclodextrin cavity, modulable biodegradability, and the biocompatibility of the component materials. Moreover, further control of biodegradability may be achieved by changing the chemical nature of the cyclodextrin-oligoesters.

## 4. Materials and Methods

### 4.1. Materials

D,L-lactide (LA—Purac, Gorkum, The Netherlands) was recrystallized from toluene, vacuum dried and stored under an Ar atmosphere in a desiccator on P_2_O_5_. β-cyclodextrin (CD—Cyclolab, Budapest, Hungary) was vacuum dried at 80 °C (0.01 mbar) for 72 h and stored in a desiccator under an Ar atmosphere on P_2_O_5_. N,N-dimethylformamide (DMF) was dried over activated 3Å molecular sieves and freshly distilled before use, isophorone diisocyanate (IPDI) and dibutyltin dilaurate (DBTL) were acquired from Sigma Aldrich (Saint Louis, MO, USA) and used as received. Polyethylene glycol (PEG, *Mn* = 2000 g/mol) from Sigma Aldrich (Saint Louis, MO, USA) was vacuum dried at 100 °C (10^−2^ mbar) for 3 h prior use to remove water traces. All other solvents, of chromatographic purity, were used as received.

### 4.2. Synthesis of Cyclodextrin-Oligolactide CDLA Derivatives

The CDLA synthesis was performed according to a previously published procedure [40,41]. In a typical synthesis procedure, 2 g of β-CD and 2 g of D,L-LA (molar ratio β-CD:D, L-LA = 1:8) and 30 mL of dry DMF were added to a round-bottomed flask with a magnetic stir bar, under the protection of Ar flow. Then, the flask was closed with a rubber septum and immersed in an oil bath on a heater with magnetic stirring at 80 °C for 72 h. The resulting compound was purified by precipitation of the crude reaction mixture in cold diethyl ether and the traces of DMF were further eliminated in a vacuum oven (20 mbar) at 50 °C for 12 h. Further, the final product was grounded leading to a fine white powder (yield 85%, structural analysis—MALDI MS and thermogravimetric analysis in Supplementary Materials).

### 4.3. Synthesis of Isocyanate Functionalized Poly(ethylene glycol) (PEG-(NCO)_2_)

In a round-bottomed flask equipped with a magnetic stirrer, 3 g of dry PEG and 6 mL of dry DMF were added under an Ar atmosphere. The mixture was stirred until an homogeneous solution was obtained. Then, 0.66 g (0.62 mL) of IPDI was added with a gas tight syringe to the reaction mixture (PEG:IPDI molar ratio of 1:2), as well as one drop of DBTL. The reaction mixture was maintained in an oil bath under magnetic stirring at 50 °C and under an Ar atmosphere. The reaction time to obtain mainly bifunctional oligomers was established at 30 min using MALDI MS (*Mn* = 2641 g/mol, *Ð* = 1.015). The obtained product was used without further purification in subsequent reactions to obtain polyurethane-cyclodextrin-based hydrogels.

### 4.4. Synthesis of CD-PEG and CDLA-PEG Hydrogels

Two types of hydrogels were prepared by reacting CDLA (G series) or β-CD (B series) as hydroxyl sources with PEG-(NCO)_2_ in different molar ratios (Table 1). In a typical procedure, solutions containing 6 mL of DMF and 3.66 g of PEG-(NCO)_2_, obtained as described in Section 4.3., were mixed with different DMF solutions of CDLA or CD in a flat-bottomed flask, under Ar. The amounts of DMF in CD or CDLA solutions were adjusted to a total concentration of 34% (weight) in the reaction mixture for each reaction. Each mixture was left for 10 min for homogenization under magnetic stirring at room temperature, after which the magnetic bar was removed, the flask was sealed and placed for 24 h in an oven heated at 50 °C. After cooling, the obtained hydrogels were transferred in an excess of acetone for 24 h (acetone was replaced twice) to remove the DMF solvent and unreacted compounds. Further, a second purification step was performed by immersion in double-distilled water (changed every 12 h) for 48 h. The swollen hydrogels were frozen at −18 °C and lyophilized to remove the water. After drying, the obtained hydrogels were weighted, kept in a desiccator, on P_2_O_5_, under an Ar atmosphere and structurally characterized.

### 4.5. Characterization Methods

#### 4.5.1. FTIR

Infrared spectra were recorded with a Bruker Vertex 70 FTIR spectrophotometer equipped with a ZnSe crystal. The FTIR spectra were registered in an attenuated total reflection in the range of 600–4000 cm^−1^, at room temperature with a 4 cm^−1^ resolution and by accumulating 32 scans.

#### 4.5.2. MALDI MS

Mass spectrometry measurements were performed using the RapifleX TOF-TOF instrument (Bruker, Bremen, Germany). The mass spectrometer was operated using FlexControl 4.0 and the acquired spectra were processed with the FlexAnalysis 4.0 software. The ionization laser power was adjusted to just above the threshold in order to produce charged species. A total of 18,000 spectra were averaged for each sample analysis. The method used to prepare the samples was dried droplet.

CDLA analysis was performed as follows: 5 mg of substance was dissolved in 1 mL acetonitrile/water mixture (volume ratio 1:1) and mixed using a Vortex Genie-2. A supersaturated solution (20 μL) of α-cyano-hydroxy-cinnamic acid (CHCA) matrix was added to 2 μL of the sample solution. Then, 2 μL of the sodium iodide (NaI) solution (5 mg/mL in methanol) was added to the sample and matrix mixture as the cations source. From the whole mixture, 1 μL was deposited on the MALDI steel plate and left to dry.

PEG-(NCO)_2_ analysis was conducted as follows: samples taken directly from the reaction mixture were rapidly introduced into 1 mL tetrahydrofuran (THF) and mixed by vibration. Then, 20 μL of trans-2-(3-(4-tert-butylphenyl)-2methyl-2-propenylidene) malononitrile (DCTB) matrix solution (20 mg/mL in THF) was added to 2 μL of then sample. Next, 2 μL of the NaI solution (5 mg/mL in THF) was added to the sample and matrix mixture. Lastly, 1 μL of the final mixture was deposited on the MALDI steel plate.

The numerical average molecular mass (*Mn*) and the gravimetric average molecular mass (*Mw*) were calculated based on the MALDI MS spectra according to Equations (1) and (2):

(1) $$Mn=\frac{\sum \frac{m_{i}}{z}a_{i}}{\sum a_{i}},$$

(2) $$Mw=\frac{\sum {\left(\frac{m_{i}}{z}\right)}^{2}a_{i}}{\sum \frac{m_{i}}{z}a_{i}},$$

where *m_i_*/*z* is the mass corresponding to the MS peak *i* and *a_i_* represent the intensity of the MS peak *i* in the mass spectrum. The molecular mass dispersity index − *Ð* of the obtained compound was also calculated as the ratio between *Mw* and *Mn* values.

#### 4.5.3. Scanning Electron Microscopy (SEM)

The surface and section morphologies of the dried hydrogels were investigated using the SEM technique. SEM micrographs were recorded using the HITACHI SU 1510 (Hitachi SU-1510, Hitachi Company, Tokyo, Japan) Scanning Electron Microscope. Samples were analyzed in surface or cross-sections as follows: the dry gels were cut transversally (parallel with the vertical axis of the cylinder) using a scalpel. The exposed transversal section was observed. The individual samples were fixed on an aluminum stub covered with a double adhesive carbon band. Before observation, the samples were placed on the chamber pedestal of a Cressington 108 Sputter Coater device and coated with a 7 nm thick gold layer, under vacuum.

#### 4.5.4. Thermogravimetric Analysis (TGA)

The thermal stability of the samples was evaluated using the Discovery TGA 2500 equipment (TA Instruments), in a nitrogen atmosphere and at a heating rate of 10 °C/min. The TG and DTG curves were processed with the TRIOS software.

#### 4.5.5. Differential Scanning Calorimetry (DSC)

DSC measurements were performed on a Discovery DSC 250 (TA Instruments), with a heating/cooling rate of 10 °C/min, under a nitrogen atmosphere. A heating-cooling-heating cycle was performed. The obtained curves were processed with the TRIOS software to identify the transition parameters.

#### 4.5.6. Rheological Characterization

Oscillatory rheological tests were carried out on a Physica MCR 501 (Anton Paar, Graz, Austria) modular rheometer equipped with a Peltier temperature control device. For all measurements, a 50 mm diameter parallel plate geometry with serrated plates was used to avoid slippage of the sample. The rheology of the CD/CDLA hydrogels was studied using oscillatory measurements for time sweep test (shear strain 5%, angular frequency 10 rad/s). All isothermal experiments were performed at a constant temperature (50 °C).

#### 4.5.7. Swelling Properties

Hydrogel samples (cut in small pieces of approximately 40–50 mg) were introduced into glass vials together with 3 mL of water and kept at a constant temperature of 23 °C. The swollen hydrogels were removed from the vials at pre-established times and the excess of liquid on their surface was gently wiped off so that they could be weighed. The weight gain, determined using an analytical balance, was monitored until the equilibrium was reached. Three different samples were measured for each hydrogel formulation.

The swelling degree (S.D.) is defined as:

S.D. (%) = (m − m_0_)/m_0_ × 100, (3)

where m and m_0_ are the swollen mass and the initial (dry) mass, respectively.

#### 4.5.8. Hydrolytic Degradation Study

Freeze-dried hydrogel samples (35–45 mg) were placed in polypropylene vials with a 1.5 mL phosphate-buffered solution (pH 7.4). The degradation of samples was carried out at 37.5 °C and each hydrogel formulation was measured in triplicate. To determine the weight loss, the samples were removed from the buffer solution at the given time, washed 3 times with distilled water and freeze dried. The weight loss (W.L.) was determined using the following formula:

W.L. (%) = m/m_0_ × 100, (4)

where m is the weight of the sample at the time of measurement and m_0_ is the initial sample weight.

## Figures and Tables

**Table 1:** Synthesis parameters of CDLA-PEG (G series) and CD-PEG hydrogels (B series).

# Sample	Molar Ratio *		Molar Ratio OH:NCO	Yields, %
	CDLA:PEG-(NCO)_2_	CD:PEG-(NCO)_2_		
G1	1:1	-	21:2	-
G2	1:2	-	21:4	-
G4	1:4	-	21:8	94
G8	1:8	-	21:16	91
G10	1:10	-	21:20	85
G12	1:12	-	21:24	82
B4	-	1:4	21:8	97
B8	-	1:8	21:16	95
B12	-	1:12	21:24	93

* Molar ratios were calculated using the *Mn* results obtained from MALDI MS.

**Table 2:** The calculated time test parameters for hydrogels.

# Sample	t_c_ (min)	G’ = G” (Pa)	lη*l (Pa·s)
B4	39.5	4.418	0.6248
B8	33.3	13.08	1.849
B12	28.3	9.174	1.297
G4	103.2	36.84	5.21
G8	86.0	20.91	2.957
G12	73.2	91.2	12.9

**Table 3:** DSC data for CD- and CDLA-PEG hydrogels.

# Sample	T_g_ (°C) ^1^	ΔC_p_^2^ (J/g·K)	T_cr_^3^ (°C Peak)	ΔH_crystallization_ (J/g)	T_melt_^4^ (°C Peak)	ΔH_melting_ (J/g)
B4	−56.1	0.48	−23.9	17.7	28.8	32.2
B8	−48.8	0.19	-	-	38.6	54.6
B12	−48.2	0.38	-	-	35.3	54.6
G4	−47.9	0.528	−0.4	27.8	32.6	28.1
G8	−51.7	0.235	-	-	39.3	46.2
G12	−51.6	0.67	−23.8	22.1	34.9	54.1

^1^ The middle of the heat capacity step. ^2^ The variation of the heat capacity during the glass transition. ^3^ Temperature of the crystallization peak. ^4^ Temperature of the melting peak.

**Table 4:** Theoretical content of the hydrogels according to synthesis feed versus degradation weight loss.

# Sample	CD/PEG Molar Ratio	Content, wt%		Weight Loss, wt%			
		CD	LA	Day			
				12	49	130	193
B8	1:8	5.5	-	0	0	0	0
G4	1:4	9.9	9.9	14	26	39	48
G8	1:8	5.5	5.5	13	18	26	36
G10	1:10	4.5	4.5	5	12	17	22
G12	1:12	3.8	3.8	3	9	11	17

## Data Availability

Not applicable.

## Supplementary Materials

The following supporting information can be downloaded at: https://www.mdpi.com/article/10.3390/gels8060348/s1, Figure S1: MALDI MS spectrum of CDLA; Scheme S1: Synthesis of PEG-(NCO)_2_ prepolymer; Figure S2: FTIR spectra of CDLA-PEG (a) and CD-PEG (b); Figure S3: The degradation curves for the CDLA precursor; Figure S4: The degradation curves for the PEG used in the synthesis; Table S1: Degradation data from TG and DTG curves; Figure S5: The thermal degradation curves of CD-PEG samples; Figure S6: The thermal degradation curves obtained for the CDLA-PEG samples; Figure S7: Dependence of swelling degree on temperature of CD-PEG (B4) and CDLA-PEG (G4) hydrogels; Figure S8: Surface micrographs for CD-PEG—(a) B8 initial, (b) B8 after 193 days, and CDLA-PEG—(c) G4 initial, and (d) G4 after 193 days; Figure S9: Surface micrographs after 193 days of degradation for CDLA-PEG: G4 at (a) 500 µm and (b) 50 µm, G8 at (c) 500 µm and (d) 50 µm, G10 at (e) 500 µm and (f) 50 µm, G12 at (g) 500 µm and (h) 50 µm.
