# Supplementary material for: The Influence of the Hydroxyl Type on Crosslinking Process in Cyclodextrin Based Polyurethane Networks
Source: Gels. 2022 Jun 2;8(6):348. doi: 10.3390/gels8060348 (PMC9222673; doi:10.3390/gels8060348)
Supplement: Supplementary file 1 [file gels-08-00348-s001.zip › gels-1741442-supplementary.pdf]

Supplementary Materials

The influence of the hydroxyl type on crosslinking process in cyclodextrin based polyurethane networks

Cristian Peptu*[1], Alexandra-Diana Diaconu[1], Maricel Danu[2], Catalina A. Peptu[2], Mariana Cristea[1], Valeria Harabagiu*[1]

[1] Petru Poni" Institute of Macromolecular Chemistry, Aleea Grigore Ghica Voda 41A, 700487 Iaşi, Romania;
[2] "Gheorghe Asachi" Technical University of Iaşi, 700050 Iaşi, Romania
* Correspondence: CP cristian.peptu@icmpp.ro; VH hvaleria@icmpp.ro

Contents:

- CDLA characterization **- Figs. S1** - page S2
- Synthesis of PEG-(NCO)$_2$ – **Scheme S1**– page S3
- FTIR spectra of hydrogels – **Fig. S3** – page S4
- The degradation curves for CDLA, PEG, hydrogels G, hydrogels B – **Figs. S3-S16, Table S31** – pages S5-S9
- Dependence of swelling degree on temperature – **Fig. S7** – page S10
- Degradation of hydrogels – **Figs. S8, S9** – pages S11, S12
- References – page S13

CDLA synthesis and characterization

In the MALDI-MS spectrum of CDLA product employed in our study, presented in **Figure 1,** there may be observed two types of peaks: a series of intense peaks, with a difference between two consecutive peaks of 144 Da, corresponding to one lactide unit (or 2 lactate units) and a second series of peaks, of lower intensity, located between the more intense peaks, at a difference of 72 Da from the neighboring peaks. The high intensity peaks represent CDLA species with *m/z = 1134 (β-CD) + n*144 (bi-lactate) + 23 (Na)* and the low intensity peaks represent also CDLA species with *m/z = 1134 (β-CD) + nx72 (lactate) + 23 (Na)*, where *n* has odd values.

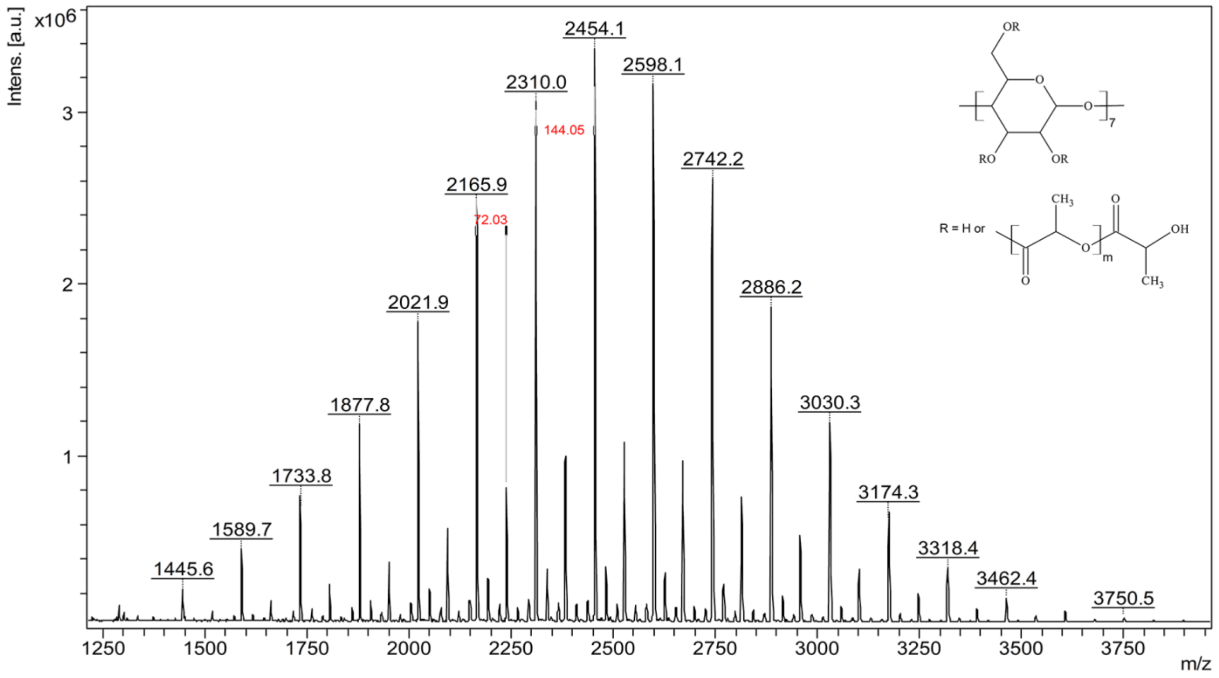

Figure S1. MALDI MS spectrum of CDLA

Based on the acquired MS spectrum it was possible to determine the numerical average molecular weight, Mn = 2443 g/mol, the gravimetric average molecular weight, Mw = 2526 g/mol, and the dispersity index, Đ = 1.033. This is corresponding to a CDLA product with approximately 8 units of LA, covalently linked to CD.

Synthesis of PEG-$(NCO)_2$

Scheme S1 shows the reaction between PEG and IPDI with the formation of the desired compound (PEG-$(NCO)_2$), 2 molecules of IPDI bound to a molecule of PEG (functionalized PEG containing 2 isocyanate groups, required for the subsequent cross linking reaction).

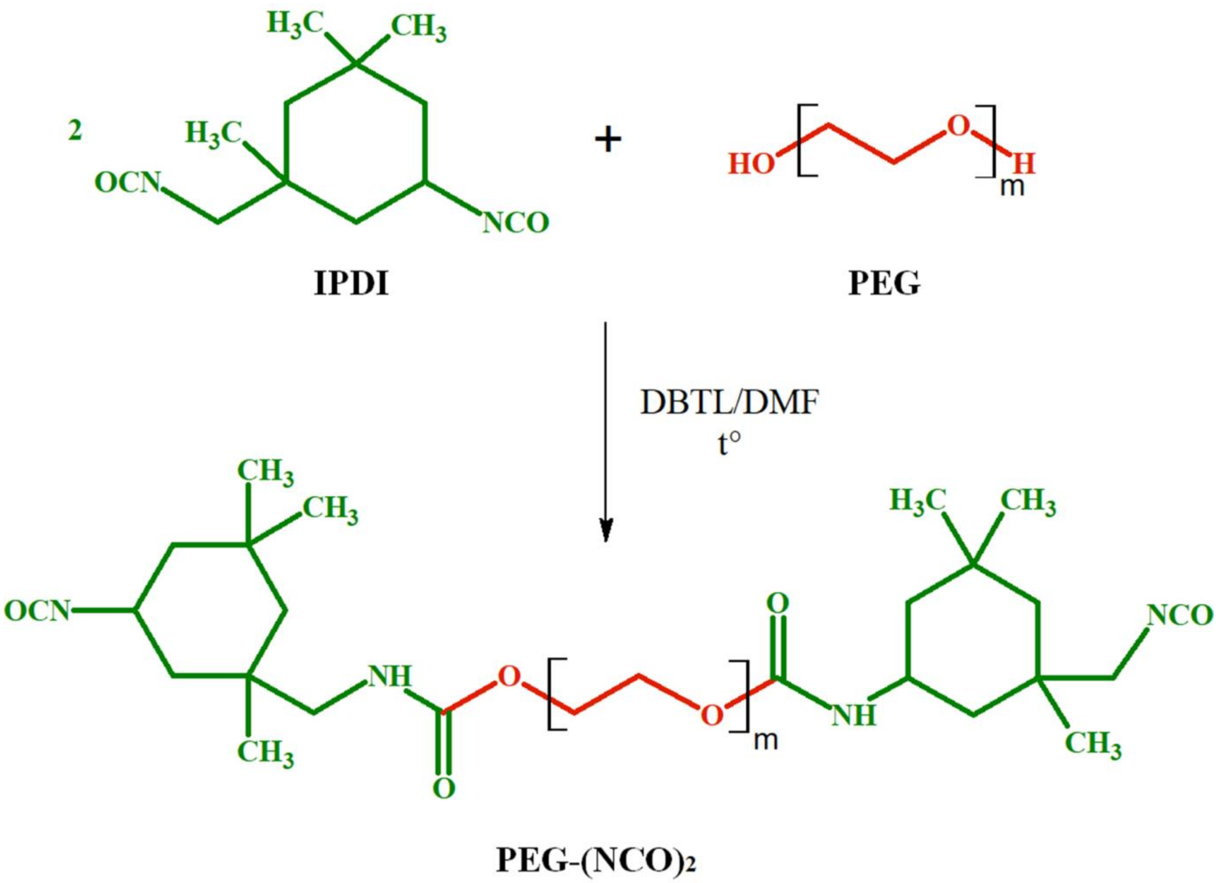

Scheme S1. Synthesis of PEG-$(NCO)_2$ prepolymer

FTIR spectra of hydrogels

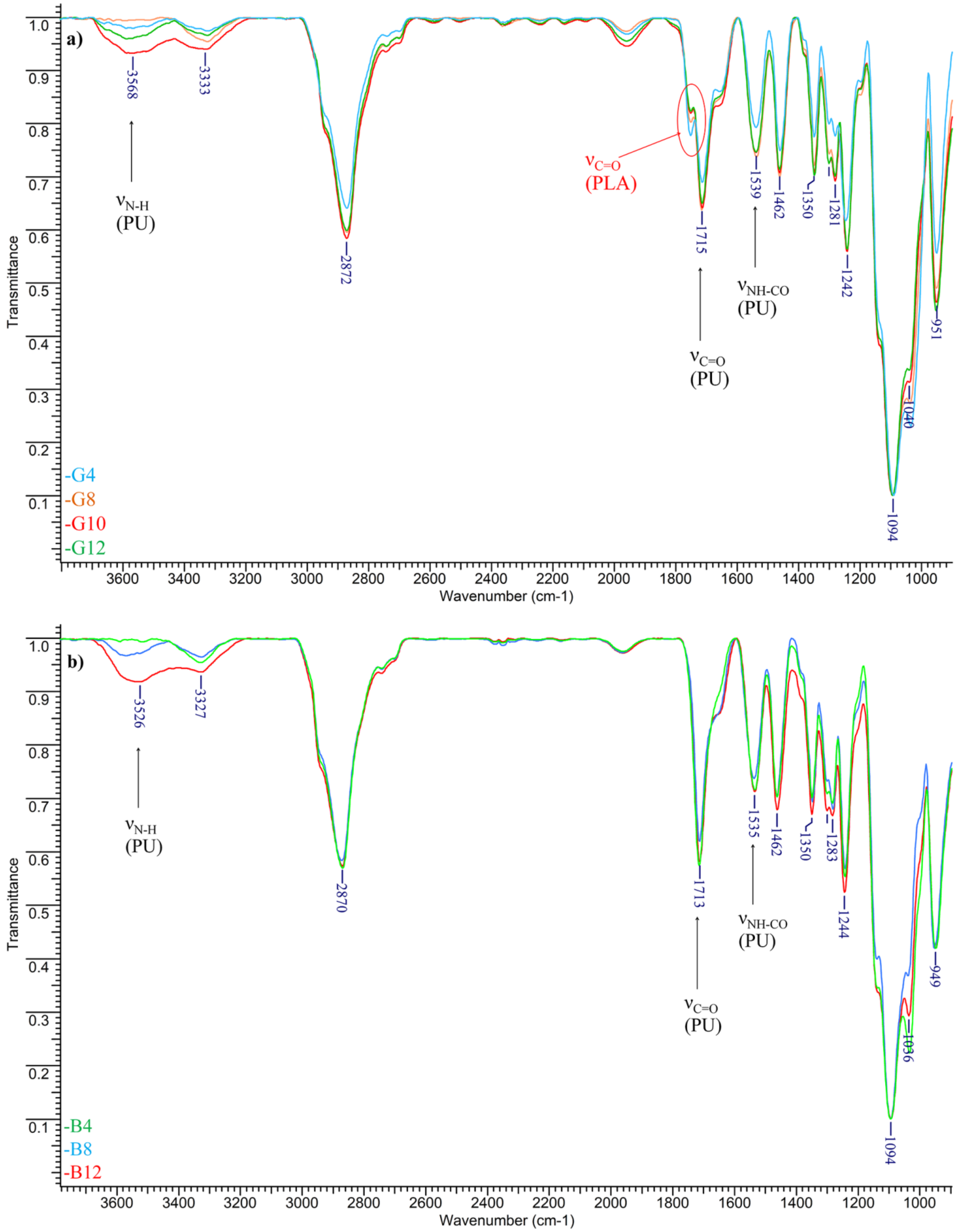

Figure S2. FTIR spectra of **CDLA-PEG** (a) and **CD-PEG** (b)

The thermal degradation curves

Usually, thermal degradation of β-CD occurs in two steps. The first region corresponds to water vaporization (60-100 °C or even above 100 °C), while the second region is attributed to the decomposition of cyclodextrin itself (T > 270 °C) [2,3]. **Figure S3** depicts the thermal behavior of the CDLA synthesized in the first stage of the reaction and details are found in the **Table S1**.

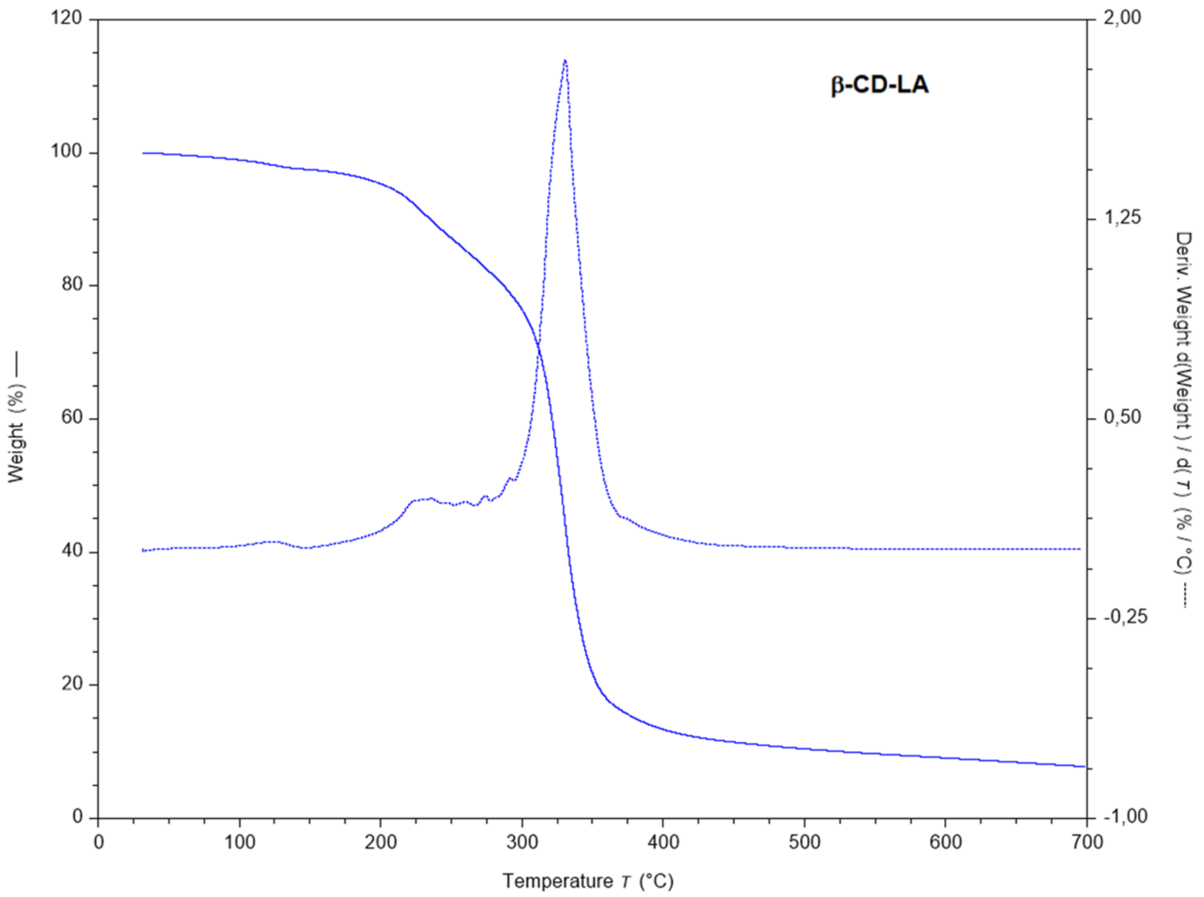

Figure S3. The degradation curves for the CDLA precursor

The onset of thermal degradation of PLA depends on its nature and processing method and is reported to be around 325 °C.

The PEG used in the synthesis starts to degrade close to 400 °C (**Figure S4, Table S1**).

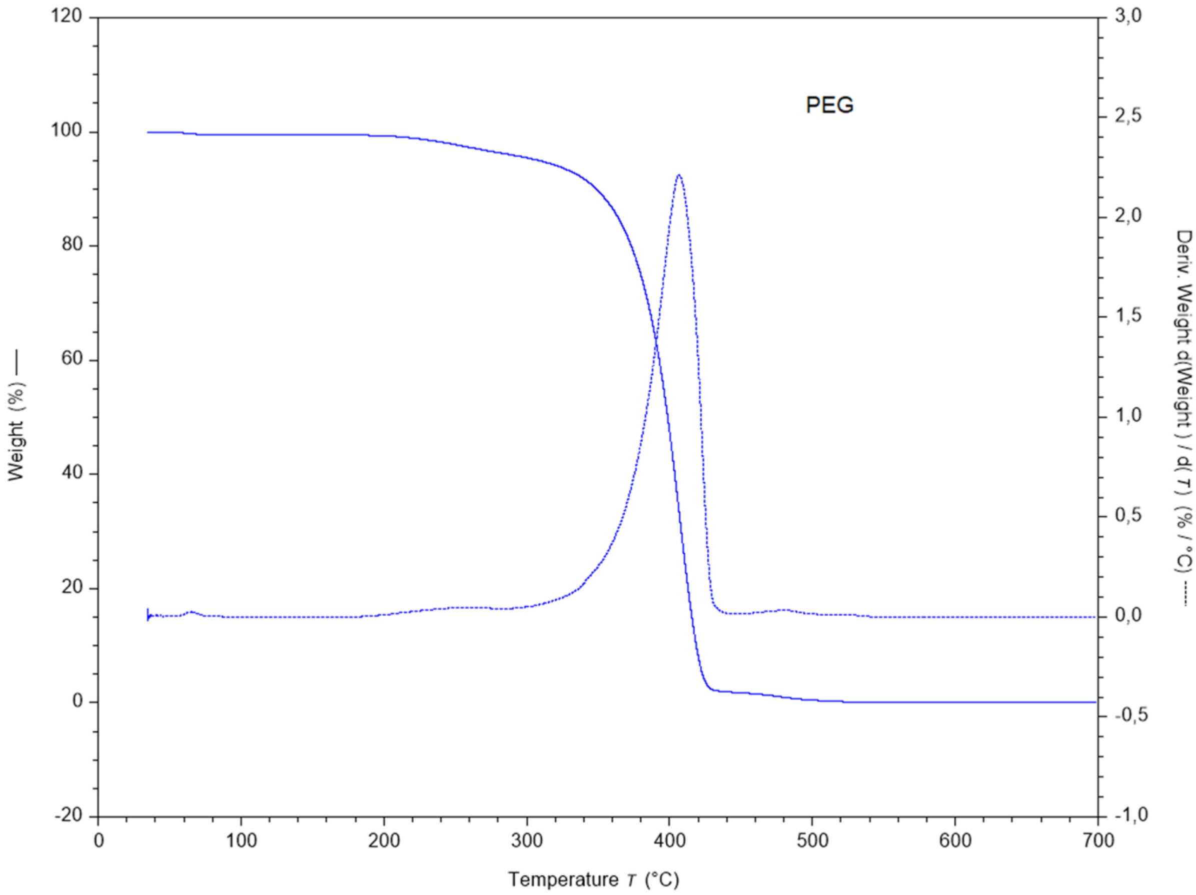

Figure S4. The degradation curves for the PEG used in the synthesis

The interpretation of the TGA spectra of the samples was done considering the above preliminary information.

Table S1 contains all the degradation data for the samples B4, B8, B12, G4, G8, G10, G12, CDLA and PEG:

- the temperature range of the degradation steps and the peak of the corresponding derivative, equivalent with the maximum rate of mass loss;
- the decomposition temperatures T_5 and T_{10}, that express the point where 5% and 10% of the total mass is lost from the sample; currently, the degradation temperature is considered to be T_5.

- the mass loss at 275 °C, 375 °C, 650 °C; the values of temperatures are chosen in order to reflect the behavior after the main degradation steps.

Table S1. Degradation data from TG and DTG curves.

#Sample	Degradation range (°C)		Peak (°C)	T_5 (°C)	T_{10} (°C)	weight loss (%) **275 °C**	**375 °C**	**650 °C**
CDLA	Step 1	190 – 250	223.1	***204.1***	235.3	17.46	84.35	91.56
	Step 2	275 - 370	330.8					
PEG	Step 1	310 - 440	329,3	***308.3***	348.7	3.41	21.18	99.99
B4	Step 1	-	-	***320.2***	342.5	1.63	20.92	97.73
	Step 2	285 - 370	345.9					
	Step 3	375 - 450	425.6					
B8	Step 1	-	-	***314.3***	334.2	1.17	18.89	98.51
	Step 2	270 - 360	335.0					
	Step 3	360 - 460	423.3					
B12	Step 1	-	-	***319.7***	343.3	1.24	18.77	99.14
	Step 2	290 – 370	351.1					
	Step 3	370 - 470	422.2					
G4	Step 1	200 – 260	249.0	***282.0***	319.2	4.37	26.1	98.16
	Step 2	267 – 375	344.4					
	Step 3	375 - 460	422.4					
G8	Step 1	190 – 245	228.2	***294.8***	323.6	3.65	22.83	98,92
	Step 2	270 – 365	346.2					
	Step 3	365 - 456	421.9					
G10	Step 1	**	-	***283.6***	316.0	4.38	25.4	98.6
	Step 2	280-361	334.0					

	Step 3	369-451	423.5					
	Step 1	**	239.4					
G12	Step 2	275 -365	337.4	***287.2***	317.0	4.06	24.4	98.52
	Step 3	365 - 450	424.7					

**faint shoulder in derivative TGA

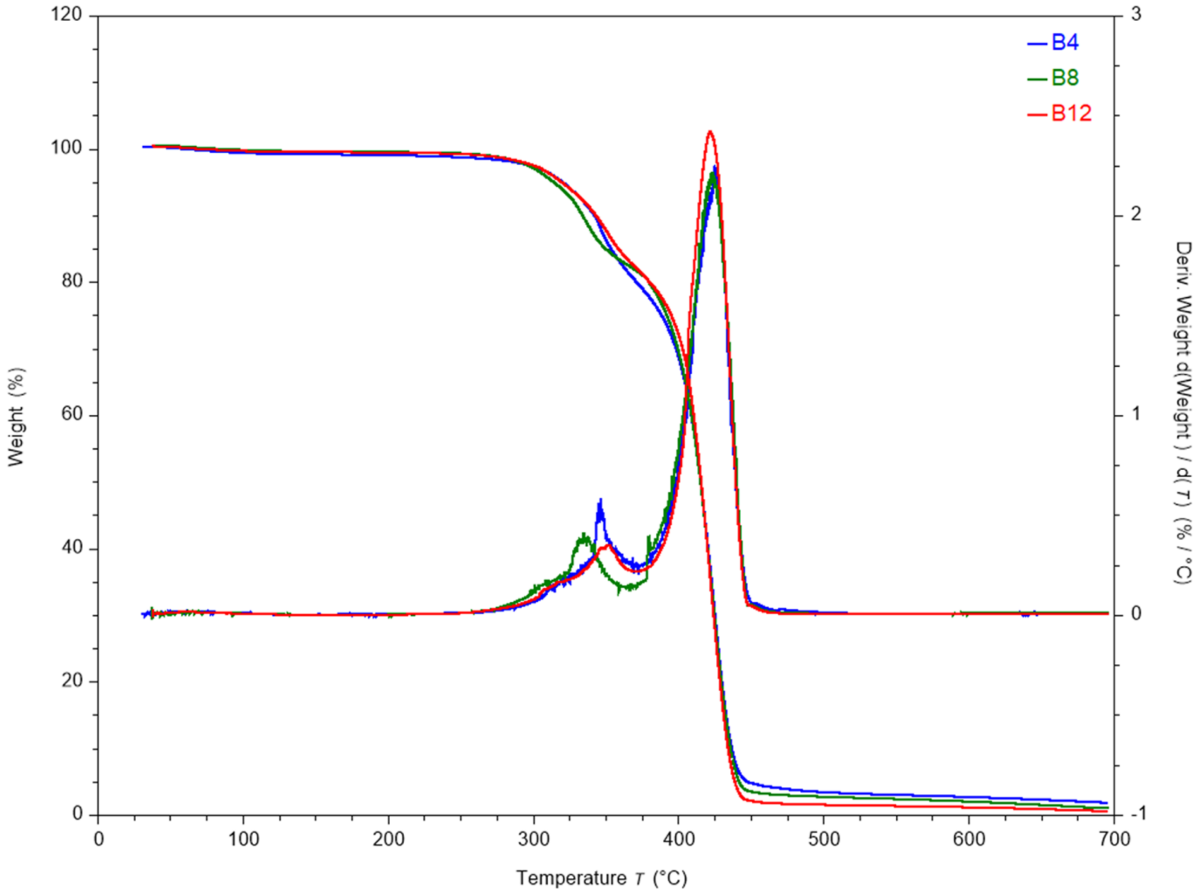

Figure S5. The thermal degradation curves of CD-PEG samples

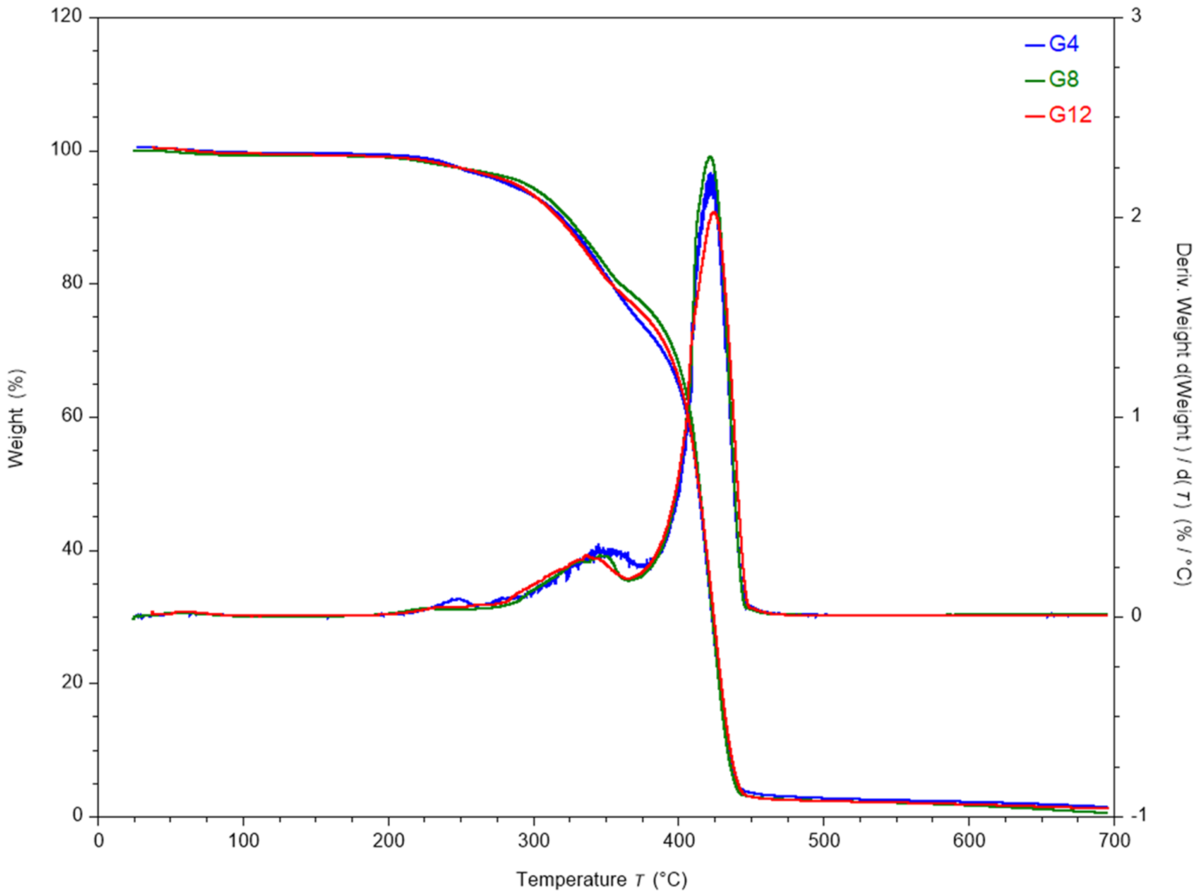

Figure S6. The thermal degradation curves obtained for the CDLA-PEG samples

Dependence of swelling degree on temperature

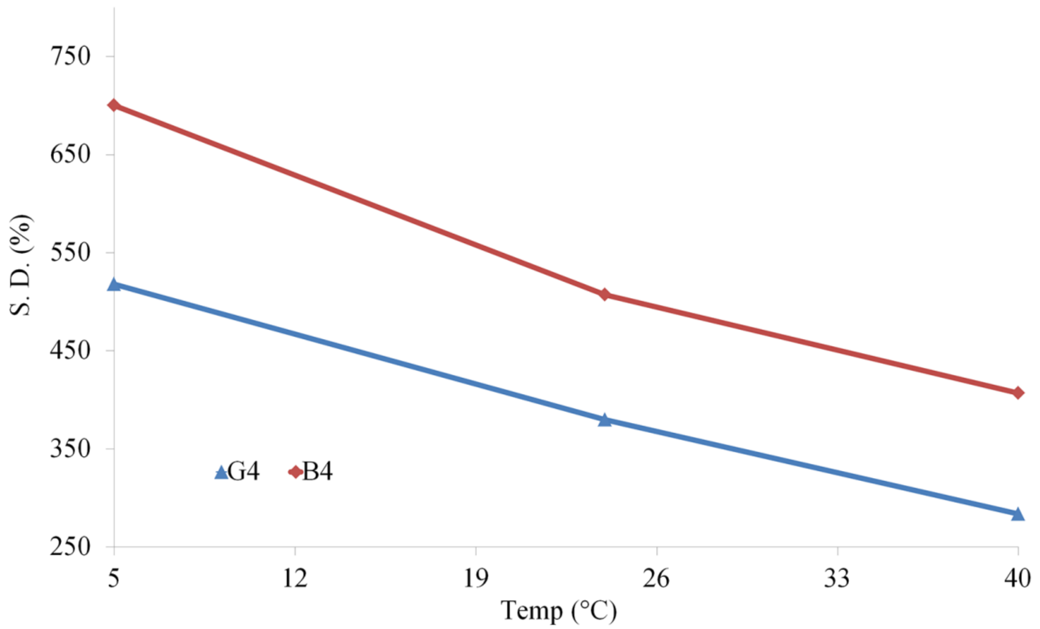

Figure S7. Dependence of swelling degree on temperature of CD-PEG (B4) and CDLA-PEG (G4) hydrogels

Hydrolytic degradation of hydrogels

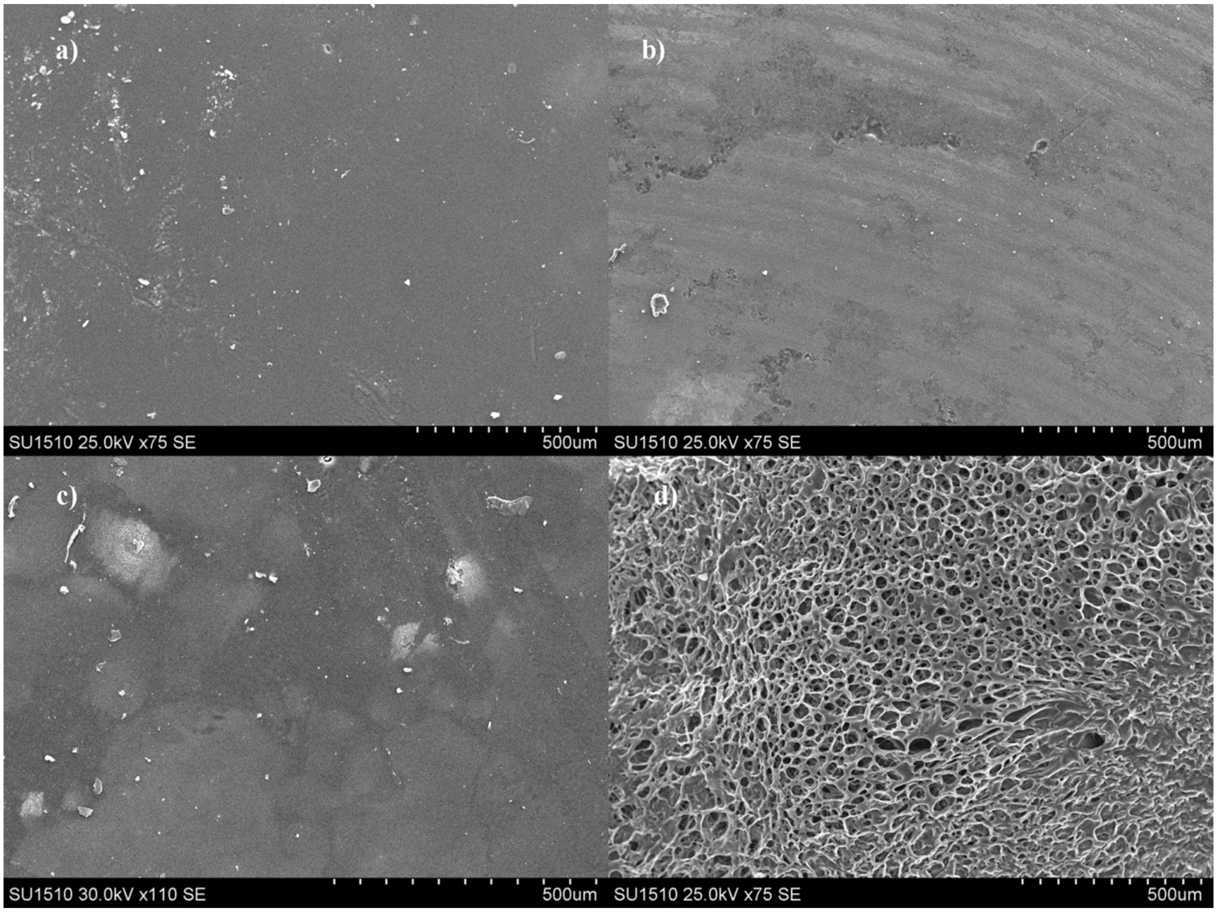

Figure S8. Surface micrographs for CD-PEG – (a) B8 initial, (b) B8 after 193 days, and CDLA-PEG – (c) G4 initial, (d) G4 after 193 days

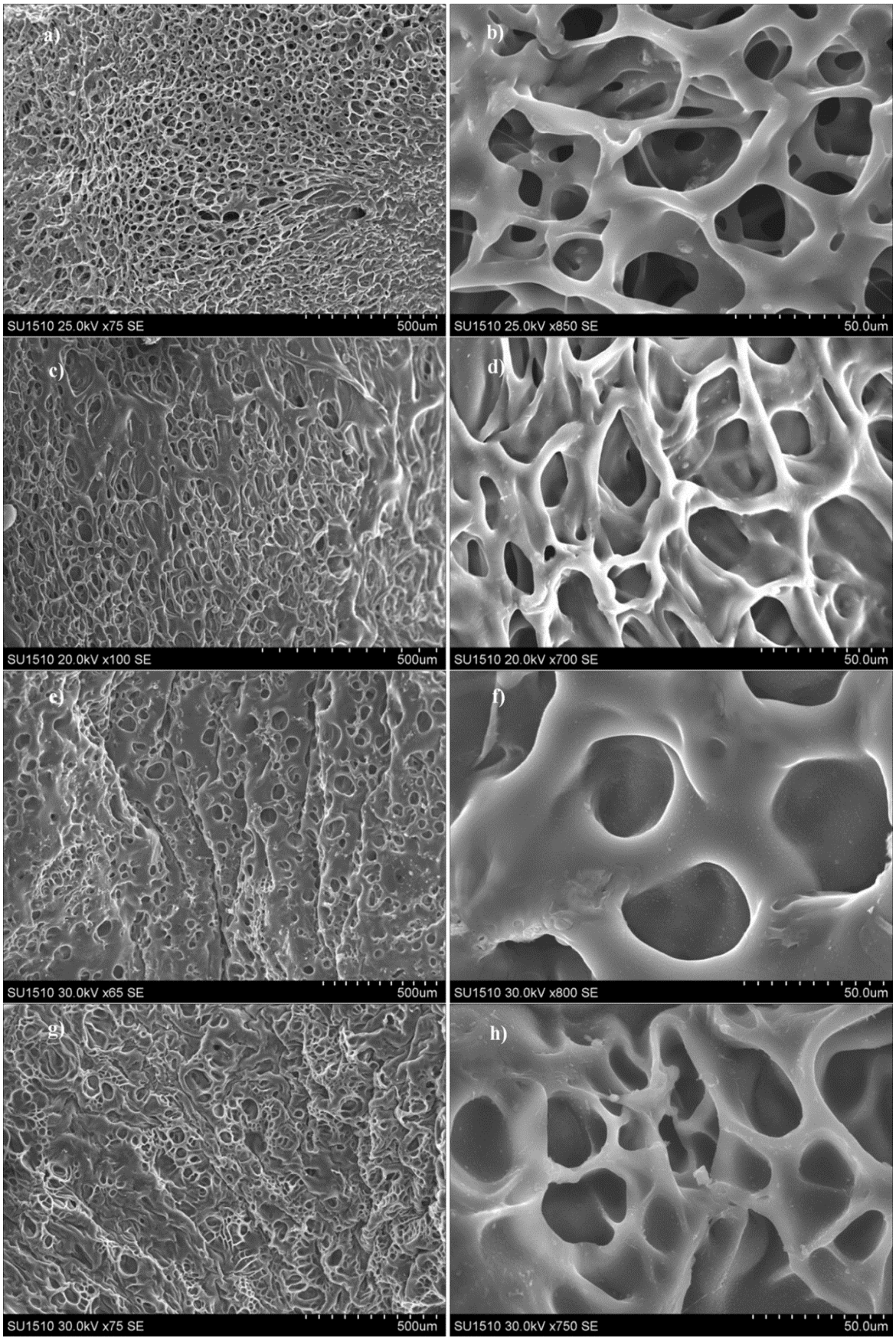

Figure S9. Surface micrographs after 193 days of degradation for CDLA-PEG: G4 at (a) 500μm and (b) 50μm, G8 at (c) 500μm and (d) 50μm, G10 at (e) 500μm and (f) 50μm, G12 at (g) 500μm and (h) 50μm

References

1. Hădărugă, N.G.; Bandur, G.N.; Hădărugă, D.I. 4. Thermal Analyses of Cyclodextrin Complexes. In *Cyclodextrin Fundamentals, Reactivity and Analysis, series Environmental Chemistry for a Sustainable World;* Fourmentin, S., Crini, G., Lichtfouse, E., Eds.; Springer International Publishing, 2018; pp. 155-221.
2. Éhen, Z.; Giordano, F.; Sztatisz, J.; Jicsinszky, L.; Novák, Cs. Thermal characterization of natural and modified cyclodextrins using TG-MS combined technique. *J. Therm. Anal. Calorim.* **2005**, *80*, 419-424.
